# Understanding the Role of Free-Living Bacteria in the Gut of the Lower Termite *Coptotermes gestroi* Based on Metagenomic DNA Analysis

**DOI:** 10.3390/insects14110832

**Published:** 2023-10-24

**Authors:** Thi Huyen Do, Trong Khoa Dao, Hong Duong Nguyen, Nam Hai Truong

**Affiliations:** 1 Institute of Biotechnology, Vietnam Academy of Science and Technology, 18-Hoang Quoc Viet, Cau Giay, Ha Noi 10000, Vietnam; khoadt2103@gmail.com (T.K.D.); duongnguyen96uet@gmail.com (H.D.N.); tnhai@ibt.ac.vn (N.H.T.); 2 Faculty of Biotechnology, Graduate University of Science and Technology, Vietnam Academy of Science and Technology, 18-Hoang Quoc Viet, Cau Giay, Ha Noi 10000, Vietnam

**Keywords:** acetogenesis, antibiotic synthesis, archaea, aromatic compound degradation, bacteria, diversity, free-living, lower termite *Coptotermes gestroi*, methanogenesis, sulfur metabolism

## Abstract

**Simple Summary:**

The lower termite *Coptotermes gestroi* is widely distributed in Northern Vietnam and is an important urban pest that destroys wooden constructions such as pagodas, temples, and furniture. Although the metagenomic DNA of prokaryotes freely living in the termite gut has been sequenced and analyzed, the overall picture of the prokaryotic diversity, including archaea and their function, has not been investigated. The present study, for the first time, revealed the differences in the structure and function of free-living prokaryotes in the termite gut. The bacterial community was formed and adapted to aid the hosts’ survival and development even in the presence of pesticides. Beyond the potential function of the bacteria towards lignocellulose digestion, free-living bacteria and archaea also harbor diverse genes coding for the enzymes/proteins involved in reductive acetogenesis, methanogenesis, methane and sulfur metabolism, and nitrogen fixation and recycling to supply energy for the host; the synthesis of antibiotics for host defense; and the detoxification of aromatic compounds. The present study provides a valuable scientific basis for the mining of novel bacterial genes and the isolation of bacteria from the termite gut for agricultural, environmental, pharmaceutical, and medical applications.

**Abstract:**

Termites’ digestive systems, particularly in lower termites with the presence of protozoa, are unique ecological niches that shelter a diverse microbiota with a variety of functions for the host and the environment. In 2012, the metagenomic DNA (5.4 Gb) of the prokaryotes that freely live in the gut of the lower termite *Coptotermes gestroi* were sequenced. A total of 125,431 genes were predicted and analyzed in order to mine lignocellulolytic genes. however, the overall picture of the structure, diversity, and function of the prokaryotic gut microbiota was not investigated. In the present study, these 125,431 genes were taxonomically classified by MEGAN and functionally annotated by the Kyoto Encyclopedia of Genes and Genomes (KEGG) and by the Carbohydrate-Active enZYmes (CAZy) and HMMER databases. As a result, 95,751 bacterial genes were classified into 35 phyla. The structure of the bacteria, typified by a high ratio of Firmicutes to Bacterioidetes, was distinct from the structure of the entirety of the bacteria in the lower or higher termites’ guts. The archaea (533 genes) were distributed into 4 phyla, 10 classes, 15 orders, 21 families, 47 genera, and 61 species. Although freely living in the guts, the prokaryotic community was formed, developed, and adapted to exhibit unique interactions in order to perform mutual roles of benefit to their hosts. Methanobacteriales, accounting for 61% of the archaea symbionts, seem to play an important role in methanogenesis. Concomitantly, bacterial methanotrophs in the gut utilize methane and combine with other bacterial groups, including potential lignocellulolytic degraders, acetogens, sulfur bacteria, and nitrogen-recycling bacteria, to efficiently convert wood with little nitrogen into acetates via certain pathway modules specified by prokaryotes that freely live in the gut. This forms an important energy source for the termites. Furthermore, bacteria carry 2223 genes involved in the biosynthesis of 17 antibiotic groups. The gut bacteria also possess genes for the degradation of 18 toxic aromatic compounds, of which four are commercial pesticides against termites commonly used for the preservation of wooden constructions. Eight of the eighteen pathways were the first to be reported from the termite gut. Overall, this study sheds light on the roles of the freely living bacteria and archaea in the *C. gestroi* gut, providing evidence that the gut microbiome acts as the second host genome, contributing both nutrients and immunity to support the host’s existence, growth, and development.

## 1. Introduction

Termites are among a few wood-feeding pests that are believed to have evolved from an ancient lineage of cockroaches. Termites belong to the infraorder Isoptera, order Blattodea, class Insecta, and phylum Arthropoda. According to the Termite database of the University of Brazil (http://164.41.140.9/catal/statistics.php?filtro=fossil, http://164.41.140.9/catal/statistics.php?filtro=extant accessed on 27 March 2023), termites comprise 3173 species, 374 genera, and 12 families, including Archeorhinotermitidae, Archotermopsidae, Cratomastotermitidae, Hodotermitidae, Kalotermitidae, Mastotermitidae, Rhinotermitidae, Serritermitidae, Stolotermitidae, Stylotermitidae, Termitidae, and Termopsidae. Based on the presence or absence of flagellate organisms in gut symbionts, termites have been split into lower and higher termites, respectively. Lower termites, which lack flagellate organisms in their guts, belong to nine families: Archotermopsidae, Hodotermitidae, Kalotermitidae, Mastotermitidae, Rhinotermitidae, Serritermitidae, Stolotermitidae, Stylotermitidae, and Termopsidae [1]. Higher termites are distinguished by the presence of fungi in their guts. Lower termites predominantly feed on wood and their capability to digest wood is based on the action of enzymes from termites and symbiotic bacteria associated with flagellates (situated inside or on the surfaces of flagellates) and attached and free-living (accounting for 7–10%) in the termite gut [2,3,4]. Higher termites generally consume a wide variety of materials, including feces, humus, grass, leaves, and roots. Lower termites and higher termites, including non-Macrotermitinae wood-feeding Termitidae, fungi-cultivating termites, and soil-feeding termites belonging to Termitidae, have drawn extensive research interest due to both their evolutionary aspects and the presence of lignocellulolytic gut bacteria [5,6,7,8]. In Asia, *Coptotermes*(Rhinotermitidae, Coptotermitinae) is one of the most important genera of wood-destroying pests [9]. In Vietnam, 141 termite species have been found, which have been classified into four families: Kalotermitidae, Termopsidae, Rhinotermitidae, and Termitidae [10,11,12]. Some species of the genus *Coptotermes* are widely distributed in all regions of Vietnam; however, *C. gestroi*, *C. emersoni*, *C. emersoni*, and *C. travians* are most frequently distributed in Ha Noi and Northern Vietnam [10,12,13]. *C. gestroi* has also been found in many places around the world, such as the Pacific Islands, North America, South America, and the islands of the Indian Ocean [14]. Due to their effective wood digestion capabilities, termites, especially *C. gestroi*, play important roles in carbon turnover and as potential sources of biochemical catalysts for the conversion of wood into biofuels. On this front, Franco Cairo et al. (2001) analyzed the digestome of the *C. gestroi* gut and identified 55 short peptide sequences within cellulolytic, xylanolytic, mannan–hydrolytic enzymes, pectinases, and starch-degrading and debranching enzymes [15]. Metatranscriptome analysis has also confirmed the role of carbohydrate-active enzymes (CAZymes) and pro-oxidant, antioxidant, and detoxification enzymes (PAD) synthesized by *C. gestroi* and the gut flagellates in the carbohydrate and lignin metabolism in termites [15]. In our previous study, 587 genes encoding 316 cellulases, 259 hemicellulases, and 12 pectinesterases and pectate lyases were observed in the *C. gestroi* gut based on KEGG database annotation of the metagenomic DNA of free-living prokaryotes [8,16]. Using the AcalPred tool [17], 59% of lignocellulolytic enzymes have been predicted to be alkaline [18]. The abundant orders and species of free-living bacteria in the termite gut have been analyzed in the past [8], but the overall picture of the bacterial diversity and the role of the free-living gut bacteria has not been fully investigated. Similarly, the archaeal diversity and function also remain largely unexplored.

The investigation of the microbiota in the termite gut has revealed that, besides the production of lignocellulolytic enzymes, gut microbes also contribute to numerous nutritional and defensive functions, such as nitrogen fixation, carbon dioxide fixation, and the recycling of many metabolites through reductive acetogenesis via the Wood–Ljungdahl pathway, methanogenesis, methane metabolism, sulfur metabolism, antibiotic synthesis, and the degradation of chemical toxins [7,19,20]. Termites play a crucial role in ecological processes and are considered potential sources of atmospheric methane, carbon dioxide, and molecular hydrogen. It is estimated that two liters of hydrogen can be produced from the digestion of one sheet of paper in the termite gut [21]. Termites are also responsible for 1% to 3% of global methane emissions [22] and can convert 42% of carbon from wood into carbonic dioxide that is emitted into the atmosphere, assimilate 18% of carbon from wood into their tissues, and release the remaining carbon (40%) into their surrounding environment as organic deposits [23]. In the gut, with high concentrations of carbon dioxide and hydrogen, the prokaryotic community in the termite gut, especially acetogens, fixes CO_2_ and reduces H_2_ to synthesize acetate by the Wood–Ljungdahl pathway to supply beneficial nutrients to the host and microbial community [24]. Moreover, a high concentration of H_2_ inhibits natural biodegradation; thus, acetogenesis also plays an important role in de-inhibiting the lignocellulose degradation in the gut [24]. On the other hand, the syntrophic interaction of methanogens with acetogens generates methane from H_2_ and CO_2_ through CO_2_ fixation by the Wood–Ljungdahl pathway. Concomitantly, sulfate-reducing bacteria oxidize acetate to H_2_ and CO_2_ by the Wood–Ljungdahl pathway in reverse, simultaneously converting sulfate to sulfide. In the termite gut, acetogens are the dominant microbial type, which is why acetate is the major energy source for the host [25]. Our previous study showed a lack of gut-flagellated protists in one termite group (TG1), indicating that the free-living bacteria in the *C. gestroi* gut are partly different from those found in the guts of other termites like *R. speratus*. Many cellulase and hemicellulase genes harboring bacteria were also observed in abundance [8]. Thus, we hypothesized that the gut prokaryotic community of *C. gestroi* composed of free-living bacteria and archaea could also carry out acetogenesis, methanogenesis, and sulfur metabolism, which might be unique and specific to the free-living prokaryotes in the termite gut.

In terms of evolution, Arora et al. (2022) sequenced 145 metagenomes of the bacterial communities in the guts of four termite groups, including lower termites (LT), soil-feeding Termitidae (SF), wood-feeding Termitidae excluding Macrotermitinae (WF), and the fungus cultivating Macrotermitinae (FC), and found that the microbial diversity and CAZyme abundance in lower termites were unique and distinct from those of the guts of other SF, WF, or FC termites [7]. However, in this paper, we hypothesize that the CAZymes from free-living prokaryotes in the *C. gestroi* gut might be closely related to those of higher termites due to the absence of flagellates.

Furthermore, evolutionary theory predicts that insects have evolved to prevent the invasion of pathogens by possessing a gut microbiota that has the capability to produce and secrete antibiotics [26]. Supporting this, many bacteria, especially Actinobacteria, producing antibiotics such as actinomycin X2, have been isolated from termite guts [26,27,28,29,30]. To our knowledge, no other pathway for antibiotic synthesis has been investigated via metagenomic studies of the termite gut microbiome, except a report of two genes encoding for carboxyl ester hydrolases capable of de-acetylating cephalosporin to semi-synthetic β-lactam antibiotics [31].

Obviously, the termite gut is a complex microhabitat having unique biotic and abiotic features, thereby providing ecological niches to functionally and ecologically diverse microbiota [32]. Many prokaryotes in the guts of lower and higher termites and cellulolytic flagellates present in lower termites are unique, as these are not found anywhere besides the termite gut [33,34,35]. Hence, the diversity and function of the prokaryotic community in the termite gut has recently emerged as a particularly intriguing topic for researchers all over the world [7,34].

From the above discussion, it is clear that complete knowledge of the role of the termite gut microbiome in many functions related to methanogenesis, methane metabolism, sulfur metabolism, antibiotic synthesis, biodegradation, etc., is still not available. Therefore, this study was undertaken to explore the functional diversity of the uncultured free-living prokaryotes in the gut of *C. gestroi,* including both bacteria and archaea, by a metagenomics approach, followed by in silico analyses.

## 2. Materials and Methods

### 2.1. Materials

In our previous study, worker termites that had destroyed a wooden pagoda and houses, from five nests in Ha Noi and one nest in Hung Yen province, Vietnam, were harvested and classified as *Coptotermes gestroi* by mtDNA 16S rRNA gene sequencing [36]. Then, the free-living bacteria of the worker termites’ guts were separated by centrifugation. Illumina de novo sequencing was used to sequence the DNA metagenome of the free-living bacteria [8]. From the obtained 5.4 Gb DNA metagenome, 125,431 putative open reading frames (ORFs, i.e., genes) were predicted, and 100,340 ORFs were classified into the bacterial superkingdom and 533 genes were classified into archaea based on the NR database and Ribosomal Database Project (RDP) from NCBI [8]. However, only 95,751 genes were classified into the phyla taxon, and only 518 of the 533 genes from archaea were distributed into the phyla taxon (Table S1). In 2012, the bacterial genes were categorized into 22 phyla, 41 classes, 97 orders, 217 families, 628 genera, and 1368 species [8]. In 2022, based on the lower taxa levels that had been designated for each gene, we retrieved the names of the phyla, thereby adding 15 more phylum names to this taxonomic profile. The archaeal genes were distributed across 47 genera and 61 species (Table S1). The profiles were used for the analysis of the free-living bacterial diversity and the function of the bacteria in the termite gut.

Previously, all the protein sequences deduced from 125,431 putative genes were subjected to BLAST against sequences available in public databases, including the Kyoto Encyclopedia of Genes and Genomes (KEGG) [8]. The sequences mapped to the KEGG metabolism pathways with E-values lower than e^−10^ and more than 50% sequence coverage were preserved [8]. This profile was used to investigate the function of the termite gut’s free-living bacteria involved in specific metabolic pathways in the termite gut.

The sequences of all 125,431 putative proteins deduced from 125,431 genes were detailed and made available in Table S1 in our previous publication [8].

### 2.2. Taxonomic Assignment

Based on the results of Nr BLAST, sequences with E-values lower than e^−10^ [8] were retrieved. The abundance of each taxonomic rank (phylum, genus, and species levels) was summarized in histograms that were drawn with Microsoft Excel 2010. To express the bacterial and archaeal diversities with the taxonomic level correlation, we used Krona, a complimentary tool in Excel (http://krona.sourceforge accessed on 27 January 2023), and drew a Krona histogram. Based on the histograms, the diversity, distribution, and abundance of each taxonomic rank were established.

### 2.3. Analysis of Diversity of Carbohydrate-Active Enzymes Producing Freely Living Prokaryotes in the C. gestroi gut

For the annotation of carbohydrate-active enzymes, all the protein sequences deduced from 125,431 putative genes were blasted against the Carbohydrate-Active enZYmes (CAZy) database (CAZy, http://www.cazy.org accessed on 15 July 2022) with threshold E-values below 10^−5^. The domains of each enzyme/protein sequence were analyzed by HMMER and PFAM, which were incorporated into the dbCAN2 database (https://bcb.unl.edu/dbCAN2/ accessed on 15 July 2022) with threshold E-values below 10^−5^. The enzymes were then categorized into glycoside hydrolases (GHs), glycosyl transferases (GTs), polysaccharide lyases (PLs), carbohydrate esterases (CEs), non-catalytic carbohydrate-binding modules (CBMs), and auxiliary activity (AAs) involved in the breakdown of lignocellulose. The abundance of the domains of each enzyme family was summarized in tables and histograms drawn with Microsoft Excel 2010.

To understand the correlations of the enzymes in the CAZy database (abbreviated as CAZymes) of the free-living prokaryotic communities with those of gut prokaryotic communities of other lower termites (LT), non-Macrotermitinae wood-feeding Termitidae (WF), fungal-cultivating termites (FC), and soil-feeding termites (SF) described by Arora et al. (2022), heatmap correlation analysis was carried out on the basis of the abundance of enzymes in the families GH, GT, PL, CBM, CE, and AA (Spearman, r ≥ 0.5, *p* < 0.05).

To elucidate the role of the gut free-living prokaryotic community in lignocellulose degradation and carbohydrate metabolism, firstly, the genes coding CAZymes were retrieved and then correlated with the taxonomy using Vlookup in Microsoft Excel. A basic map illustrating the correlations of abundant bacteria at different classification taxa with CAZyme genes was drawn using Microsoft Excel 2010.

### 2.4. Analysis of the Role of the Termite Gut Prokaryotes in Important Metabolic Pathways

Based on the results of KEGG functional annotation, the genes coding for proteins/enzymes related to the Wood–Lijungdahl pathway, reductive acetogenesis, methanogenesis, methane metabolism, sulfur metabolism, nitrogen recycling, antibiotic synthesis, and the biodegradation of certain chemicals were retrieved separately. Using the gene codes, the taxonomical information of the genes was extracted from the taxonomic profile using the Vlookup function in Microsoft Excel 2010. The enzymes participating in a specific pathway were demonstrated in a drawn figure, and the abundance of each taxonomic rank (phylum, genus, and species levels) was summarized in histograms drawn with Microsoft Excel 2010. The relative abundance of each taxonomy level was summed. The taxonomic level correlation was drawn using the Krona tool in Excel.

## 3. Results

### 3.1. Overall Gut Microbial Diversity

Free-living prokaryotes in the *C. gestroi* termite gut included both eubacteria (i.e., bacteria) and archaea. However, as revealed by the metagenomic analysis, the bacterial genes accounted for 80% of the total genes, while archaeal genes only accounted for 0.42%. In 2012, the bacterial genes were categorized into 22 phyla, 41 classes, 97 orders, 217 families, 628 genera, and 1368 species [8]. However, in 2022, based on the lower taxa levels designated for each gene, we retrieved the phyla names and added 15 more phyla, namely Actinobacteria, Thermotogae, Deferribacteres, Ignavibacteria, Elusimicrobia, Calditrichota, Mycoplasmatota, Aquificae, Cloacimonetes, Pseudomonadota, Chrysiogenetes, Lentisphaerota, Gemmatimonadetes, Campylobacterota, and Thermodesulfobacteria; thus, a total of 95,751 bacterial genes were classified into 35 phyla, 54 classes, 115 orders, 242 families, 661 genera, and 1279 species. The archaea were distributed into 4 phyla, 10 classes, 15 orders, 21 families, 47 genera, and 61 species, with a total of 533 genes (Table S1).

**Figure 1:**
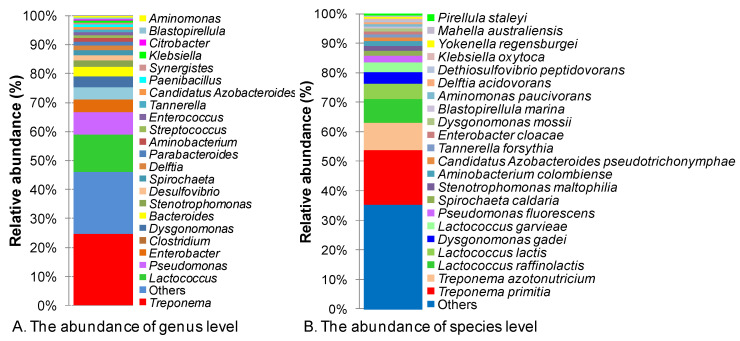
Histograms showing the abundance distribution of bacterial community genus (**A**) and species (**B**) levels.

In lower termites, bacteria and archaea have been observed both inside and on the surfaces of flagellate cells [37]; however, our main focus was free-living prokaryotes including bacteria and archaea in the termite gut. Accordingly, Firmicutes was found to be the most abundant phylum, accounting for 29% of the total 95,751 genes that were classified into phyla from a total of 100,310 bacterial genes, followed by Proteobacteria (23%), Spirochaetales (23%), Bacteroidetes (15%), and Synergistetes (6%) (Table S2). Meanwhile, Fibrobacteres only accounted for 0.02% of the total diversity. In the class taxon, Spirochaetia was the most dominant class, accounting for 23% of the total diversity, followed by Gammaproteobacteria (18%), Bacilli (15%), Bacteroidia (13%), Clostridia (12%), and Synergistia (4%). Among the seven most abundant classes, Spirochaetia had the greatest abundance (accounting for 23%) but the lowest diversity, consisting of only a single order, Spirochaetales, followed by Lactobacillales (14%), Bacteroidales (13%), Clostridiales (10%), Enterobacteriales (9%), and Pseudomonalales (6%). Corresponding to the order of abundance, seven eubacterial families accounted for 63% of the genes, namely Spirochaetaceae (23%), Streptococcaceae (12%), Enterobacteriaceae (9%), Pseudomonadaceae (6%), Porphyromonadacease (6%), Synnergistaceae (4%), and Clostridiaceae (3%) (Figure S1). Of the 95,751 genes from classified bacteria, only 75,645 genes (79%) were categorized into 661 genera (Table S2 and Figure S1). The most abundant genus was *Treponema*, which accounted for 24.7% of 75,645 classified genes. Lesser genes were assigned to the genera *Lactococcus* (12.9%), *Pseudomonas, Enterobacter*, *Clostridium*, *Dysgonomonas*, *Bacteroides*, *Stenotrophomonas*, and *Desulfovibrio* (Figure 1A). At the species level, only 55,614 genes were classified into 1279 species. A significant proportion, amounting to 41.9%, remained unclassified. The seven most abundant species, *T. primitia, T. azotonutricium, L. raffinolactis, L. lactis, D. gadei, L. garvieae,* and *P. fluorescens*, represented 50.4% of the classified genes (Figure 1B).

### 3.2. Diversity of Free-Living Archaea in the Termite Gut

**Figure 2:**
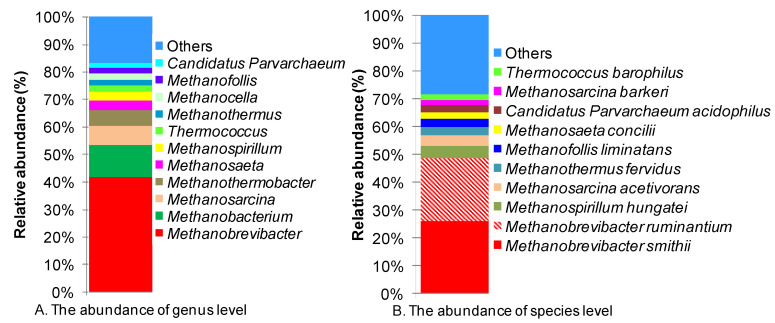
Histograms showing the abundance distribution of archaeal community at genus (**A**) and species (**B**) levels.

Using the NR database, 533 genes from the metagenomic libraries could be affiliated with archaea, whereas only 518 genes were assigned to the phylum taxon. However, archaea in the studied metagenome were low in diversity, which was clear from the fact that Euryarchaeota alone accounted for 97% of the genes and only a small proportion of genes belonged to Crenarchaeota (2%), Thaumarchaeota (1%), and Korarchaeota (0.2%) (Figure S2). In the class taxon, Methanobacteria accounted for 61% of genes, followed by Methanomicrobia (20.3%), Halobacteria (3.6%), and Themococci (3.4%). The diversity of the class Methanobacteria was rather low, with representation from only one order, Methanobacteriales. Contrastingly, the class Methanomicrobia contained three abundant orders, including Methanosarcinales, Methanomicrobiales, and Methanocellales. At the family level, Methanobacteriaceae was the most abundant, with 58% of genes, followed by Methanosarcinaceae (7.1%), Halobacteriaceae (3.6%), Thermococcaceae (3.4%), Methanomicrobiaceae (3.0%), Methanosaetaceae (2.6%), Methanospirillaceae (2.6%), Methanothermaceae (1.9%), Methanocellaceae (1.7%), and Parvarchaeaceae (1.5%) (Table S3). At the genus and species levels, a significant number of genes remained unclassified, corresponding to 18.4% and 40.3% of the total genes, respectively. *Methanobrevibacter* was the most dominant genus, holding 34.1% of the total archaeal genes, corresponding to 41.8% classified genes (Figure 2A). The genera *Methanobacterium, Methanosarcina, Methanothermobacter, Methanosaeta,* and *Methanospirillum* had lower abundance. Meanwhile, at the species level, the six most abundant species, *M. smithii*, *M. ruminantium*, *M. hungatei*, *M. acetivorans*, *M. fervidus,* and *M. liminatans,* all belonged to a single phylum, Euryarcheaota (Figure S2). The species took 37.5% of the total archaeal genes, corresponding to 62.9% of the classified genes (Figure 2B).

### 3.3. The Carbohydrate-Active Enzymes of Prokaryotes Free-Living in the C. gestroi Gut

To investigate the function of free-living prokaryotes in the gut of the lower termite *C. gestroi*, we identified genes coding for carbohydrate-active enzymes (CAZymes) using Hidden Markov model searches against the dbCAN2 database, accessed on 15 July 2022 [38]. In total, we found 2175 domains/2165 genes belonging to bacteria, which were annotated to 1300 GHs (that hydrolyzed and/or rearranged glycosidic bonds in polysaccharides), 554 GTs (that were involved in the formation of glycosidic bonds), 26 PLs (that were responsible for the non-hydrolytic cleavage of glycosidic bonds), 172 CEs (that hydrolyzed carbohydrate esters), 22 AAs (that were redox enzymes acting in conjunction with CAZymes), and 101 CBMs (that assisted enzymes in binding to their substrates) (Table 1).

Among the six CAZyme families, CBMs and AAs play an important role in the deconstruction of lignocellulose by loosening the cellulose fibers and oxidizing the cellulose in the crystalline region, thereby improving the accessibility of the cellulose to celluases and enabling the efficient decomposition of recalcitrant lignocelluloses. The most abundant CBM in the present study was CBM67, which was found to collocate with GH78, having alpha-L-rhamnosidase activity. Almost all CBMs found in this study, such as CBM32, CBM48, CBM62, CBM13, CBM6, CBM51, CBM9, CBM20, CBM35, CBM22, CBM5, CBM50, and CBM66, are capable of binding to cellulose and hemicellulose in the lignocellulose structure, thereby contributing to the effective digestion of lignocellulose. Some other CBMs were also observed, such as CBM34 and CBM41, which can bind to pullulan; CBM73, which can bind to chitin; and CBM77, which can bind to pectin (Table S4). The presence of such diverse CBMs could be attributed to the wood-feeding nature of the lower termite *C. gestroi*. In the AA family, AA4 was the most abundant and is responsible for the conversion of a wide range of phenolic compounds bearing side chains at the para-position of the aromatic ring. AA10 (formerly CBM33) was the second most dominant AA, representing the copper-dependent lytic polysaccharide monooxygenase (LPMO) activity. Some enzymes of AA10 are also known to act on chitin. LPMO is an important and powerful redox enzyme whose presence enhances lignocellulose deconstruction during enzymatic hydrolysis. The other AAs are known to be involved in the degradation of several chemical compounds, in addition to the breakdown of lignocellulose.

Of the 12 CE families, CE10 was the most abundant, and it is known to typically collocate with xylanase GH43 for xylan hydrolysis. Enzymes belonging to GH are the most diverse, with 85 different families known to hydrolyze different polysaccharides, such as cellulose, hemicellulose, starch, bacterial mucin, pullulan, chitin, lipopolysaccharide, etc., but their major role is in lignocellulose degradation, as reported earlier [8].

The investigation of bacteria harboring CAZymes revealed that the five most abundant bacterial phyla were also the most abundant phyla bearing the enzymes. The most abundant (32.5%) phylum was Firmicutes, followed by Proteobacteria (14.8%), Bacteroidetes (12.5%), Spirochaetes (11.6%), and Synergistetes (3.2%). While Firmicutes possess GHs (31.8%), GTs (31.4%), CEs (43.3%), CBMs (32.6%), and AAs (36.4%) in high abundance, PLs are the least abundant. Bacteroidetes contributed mostly CBMs (42.1%) and only a few GHs (8.4%). Protobacteria harbored a major proportion of AAs (54.5%) and contributed ~40% PLs, 20% CEs, 20% GTs, ~12% GHs, and a few CBMs (Figure S3). Thus, it can be concluded that the Protobacteria may participate in other mechanisms, in addition to polysaccharide degradation (Figure S3). At the family taxon level, Spirochaetaceae, Streptococaceae, and Enterobacteriaceae harbored genes coding for all CAZyme groups, including GHs, GTs, CEs, CBMs, PLs, and AAs. Notably, Bacteroidaceae contributed the maximum genes coding for enzymes GHs, CBMs, and PLs, helping to digest the crude substrates derived from lignocellulose (Figure S3).

**Figure 3:**
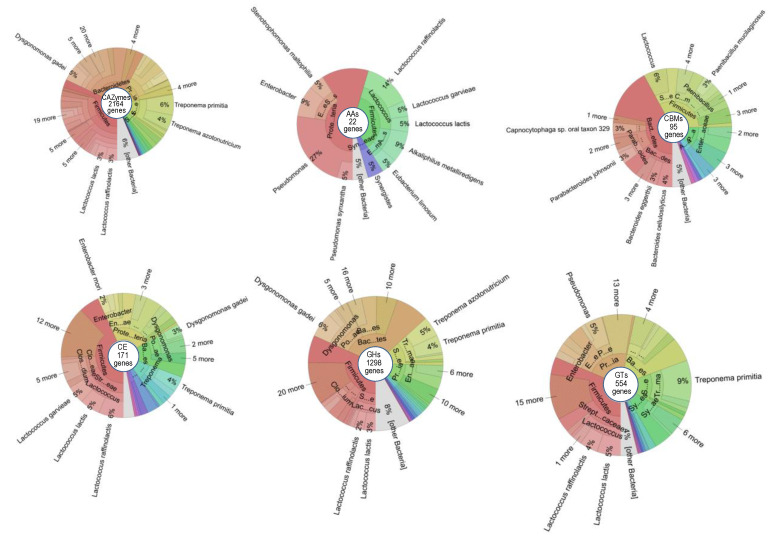
The compositional distribution of bacteria harboring CAZymes in *C. gestroi* gut.

At the genus level, there was a significant part of the genes that remained unclassified, accounting for 18.2% to 33.7% of the genes depending on the six groups of CAZymes (Figure S3). The proportion of unclassified genes at the species taxon increased, ranging from 33.3% to 47.5% depending on the CAZyme group. *Treponema*, *Lactococcus*, *Dysgonomonas*, *Bacteroides*, *Pseudomonas*,and *Clostridium* play an essential role in polysaccharide metabolism, because 54.5% of the classified genes came from these genera. *T. primitia*, *T. azotonutricium*, *D. mossii*, *E. faecalis*, *M. australiensis*, and *A. colombiense* harbored 22.3% of the classified genes coding for GHs, GTs, and CEs but did not harbor any genes for CBMs or AAs. Thus, these species have potential for the degradation of simple carbohydrate fibers. In contrast, *D. gadei, L. lactis, L. raffinolactis,* and *L. garvieae* supported 26.8% of the classified genes coding for GHs, GTs, CEs, CBMs, and AAs, indicating their strong potential for lignocellulose degradation in the termite gut. *Bacteroides cellulosilyticus*, in particular, bore GHs and CBMs, indicating this species as a good candidate for lignocellulose deconstruction and degradation (Figure S3 and Figure 3).

### 3.4. Diversity and The Role of Freely Living Gut Prokaryotes in Reductive Acetogenesis

**Figure 4:**
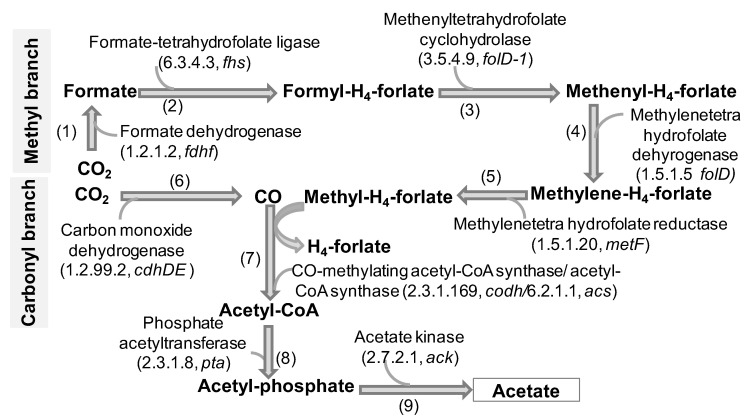
The KEGG annotated enzymes involved in the Wood–Ljungdahl pathway of acetogenesis that were mined from metagenomic DNA data of freely living prokaryotic community in *C. gestroi* gut.

In the termite gut, wood fibers are digested and fermented into H_2_, CO_2_, and acetate. Acetate is an important energy source for the growth and development of the host. Moreover, in CO_2_, hydrogen sinks, and acetogenic bacteria in the termite gut reduce excess CO_2_ using H_2_ as the electron donor to produce additional acetate by the Wood–Ljungdahl pathway (WLP). Therefore, to investigate the function of free-living prokaryotes involved in reductive acetogenesis in the *C. gestroi* gut, we mined the genes *fdhF, fhs, folD-1, folD, metF, cdhDE, codh, acs, pta,* and *ack* encoding for formate dehydrogenase (EC 1.2.1.2), formate-tetrahydrofolate ligase (EC 6.3.4.3), methenyltetrahydrofolate cyclohydrolase (EC 3.5.4.9), methylenetetrahydrofolate dehydrogenase (NADP+)(EC 1.5.1.5), methylenetetrahydrofolate reductase (NADPH)(EC 1.5.1.20), carbon monoxide dehydrogenase (EC 1.2.99.2), CO-methylating acetyl-CoA synthase (EC 2.3.1.169), acetyl-CoA synthetase (EC 6.2.1.1), phosphate acetyltransferase (EC 2.3.1.8), and acetate kinase (EC 2.7.2.1), respectively, participating in the WLP [39]. In total, 376 genes encoding for one set of the ten enzymes in the WLP were retrieved, of which *fdhF* genes were the most abundant (102 genes), followed by *acs*. The enzymes were distributed in the two branches of WLP, the methyl branch and carbonyl branch, both initiated from CO_2_ (Figure 4). The methyl branch consists of five steps to generate a methyl active group. The carbonyl branch consists of only one step to release CO. The methyl active group from the methyl branch is used for the methylation of CO, followed by CO-methylating acetyl-CoA synthase and acetyl-CoA synthetase activation to synthesize acetyl-CoA. Acetyl-CoA enters step 8 and then step 9 of the WLP to produce acetate (Figure 4).

These 10 enzymes were found to be distributed in eight bacterial phyla and one archaeal phylum (Table S5). Among them, Firmicutes represented 25.5% of the total genes, Spirochaetes supplied 25% of the genes, and Proteobacteria gave 19% of the genes in the WLP. Phyla with lower abundance were Bacteroidetes (9.6%) and Synergistetes (6.9%). Regarding abundant phyla, nine genera, including *Treponema*, *Lactococcus*, *Desulfovibrio*, *Clostridium*, *Dysgonomonas*, *Pseudomonas*, *Enterobacter*, *Candidatus Azobacteroides*, and *Dethiosulfovibrio*, predominated in harboring WLP genes. *Treponema*, *Lactococcus*, *Desulfovibrio*, *Clostridium*, and *Dysgonomonas* were identified as some potentially acetogenic bacterial genera because of the presence of all enzymes involved in the WLP. However, *Enterobacter* and *Dethiosulfovibrio* might have lower acetogenic potential because *Dethiosulfovibrio* biased the enzymes involved in steps 1 and 9, and *Enterobacter* favored enzymes in steps 1, 5, and 7; thus, these genera lacked many enzymes in the WLP (Figure 4). At the species taxonomic level, *T. primitia, L. raffinolactis,* and *T. azotonutricium* were the most abundant, with the highest gene numbers mined (57, 19, and 14 genes, respectively) (Table S6). However, none of the species covered all enzymes mined in this study for the WLP. *T. primitia* and *L. raffinolactis* harbored the largest numbers of enzymes involved in the WLP, while *T primitia* lacked the enzyme for step 6, and *L. raffinolactis* lacked the enzymes for steps 2 and 6. The investigation of potentially acetogenic bacteria on the basis of the ratio of total WLP enzymes to total WLP steps revealed that *L. lactis*, *C. Azobacteroides* pseudotrichonymphae*, D. gadei*, *L. garvieae*, and *S. caldaria* were the best potential candidates. Notably, in *L. lactis*, all six enzymes were found to participate in six steps of the WLP.

### 3.5. The Role of Freely Living Gut Prokaryotes in Methanogenesis

The methanogenic pathway consists of a pathway synthesizing methane from H_2_/CO_2_ (hydrogenotrophic pathway) and a pathway producing methane from acetate (acetotrophic pathway). Methane is the final product of the methanogenesis pathway, involving the activity of the methyl-coenzyme M reductase complex (*mcr*) in the final step of methanogenesis in symbiotic methanogenic archaea. From the metagenomic DNA data, based on KEGG annotation, we found six genes of *mcr*ABDG encoding for methyl-coenzyme M reductase. All the genes came from the order Methanobacteriales, family Methanobacteriaceae, and were distributed into two genera, *Methanobacterium* and *Methanothermobacter*. One gene was identified as the origin of *M. smithii*. This species was observed to be the most abundant in the free-living prokaryotic DNA extracted from the *C. gestroi* gut. However, *mcr*ABG was not found to have originated from the bacterial community in this study (Table S6). Besides the *mcr* gene, we also observed one *frc*A gene, eight *hdr*ABC genes, six *mtr*ABCDEF genes, three *phgdh* genes, and a *psphd* gene, respectively, encoding for methanogenic-participating enzymes including coenzyme F420 hydrogenase (EC 1.12.98.1), heterodisulfide reductase (EC 1.8.98.1), tetrahydromethanopterin S-methyltransferase (EC 2.1.1.86), D-3-phosphoglycerate dehydrogenase (EC 1.1.1.95), and phosphoserine phosphatase (EC 3.1.3.3) (Table S6). While FRCA, HDRABC, and MTRABCDEF also participated in the hydrogenotrophic pathway as MCRABDG, all the genes coding for enzymes involved in the hydrogenotrophic pathway were derived from the Methanobacteriaceae family and classified into three genera, *Methanobrevibacter, Methanothermobacter,* and *Methanobacterium*. Two *hdr*A genes were found to be from *M. smithii*, two *hdr*B and one *hdr*C were derived from *M. ruminantium*, and two *mtr* genes originated from *M. smithii* and *M. ruminantium* species.

### 3.6. The Role of Freely Living Gut Prokaryotes in Methane Metabolism

Archaea in the lower termite have many genes associated with the methanogenesis pathway to produce methane, which is one of the most important gases leading to the greenhouse effect. In the lower termite, the presence of methane-utilizing bacteria (methanotrophs) can assist in controlling methane emissions into the atmosphere. Methanotrophs are capable of methane oxidation through the host enzyme methane monooxygenase. In this study, we found 384 genes that encoded enzymes involved in methane metabolism, of which four genes were found to belong to archaea, 356 genes to bacteria, and 24 genes remained unclassified. The archaeal genes participating in formaldehyde assimilation and the ribulose monophosphate pathway convert methanol from methane into D-fructose-6-P (Table S7). These genes were also distributed in bacteria. However, the key enzymes, such as EC 1.14.3.25 or EC 1.14.18.3, which are involved in the catalytic conversion of methane into methanol, were not found in this study.

### 3.7. The Role of Freely Living Gut Prokaryotes in Sulfur Metabolism

**Figure 5:**
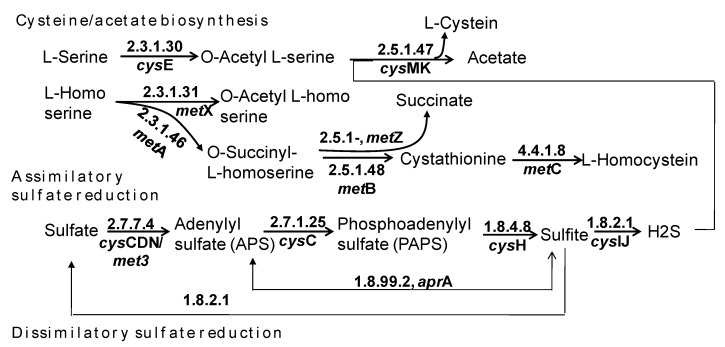
The KEGG-annotated enzymes involved in sulfur metabolism that were mined from metagenomic DNA data of prokaryotic community freely living in *C. gestroi* gut.

Sulfate-reducing bacteria operate the Wood–Ljungdahl pathway in the reverse direction and carry out the oxidation of acetate to CO_2_ and H_2_, with the concomitant reduction of sulfate to sulfide to generate energy. Therefore, such bacteria are of great significance in the final step of the Wood–Ljungdahl pathway in carbon and sulfur recycling. In this study, we found 316 genes coding for 14 enzymes involved in sulfur metabolism and only two genes came from archaea, while 310 genes had a bacterial origin (Table S8). Sulfur metabolism is divided into four pathway modules, including assimilatory sulfate reduction, dissimilatory sulfate reduction, thiosulfate oxidation by SOX, and cysteine/acetate biosynthesis. Of the four pathway modules, we found three modules in the free-living prokaryote community of the *C. gestroi* gut, with the absence of thiosulfate oxidation by SOX (Figure 5). In the branch producing L-cysteine, O-acetyl-L-serine combined with hydrogen sulfide to generate L-cysteine and acetate based on the catalysis of O-acetyl-L-serinesulfhydrylase (EC 2.5.1.47).

A total of 226 genes were found to be the most abundant genes involved in cysteine/acetate biosynthesis, accounting for 71.5% of the genes involved in sulfur metabolism. This means that the free-living bacteria in *C. gestroi* played an important role in the conversion of sulfate to energy, typically in the form of acetate, for the host. On the other hand, the numbers of genes encoding enzymes participating in assimilatory sulfate reduction were much higher than those for enzymes participating in the dissimilatory sulfate reduction module (Table S8). The released H_2_S from assimilatory sulfate reduction would contribute to cysteine/acetate production (Figure 5). Of the two genes from archaea, one gene encoded for serine O-acetyltransferase (EC 2.3.1.30), involved in cysteine/acetate biosynthesis, and one gene encoded for phosphoadenosine phosphosulfate reductase (EC 1.8.1.2), participating in assimilatory sulfate reduction.

The evaluation of the role of bacteria in sulfur metabolism revealed nine phyla harboring genes for sulfur metabolism, predominated (representing 94.2% of classified genes) by Spirochaetes, Proteobacteria, Firmicutes, and Bacteroidetes. Meanwhile, Proteobacteria and Bacteroidetes might be involved in all three pathway modules of sulfur metabolism, but Firmicutes and Spirochaetes comprised genes only for cysteine/acetate production and assimilatory sulfate reduction (Table S8). At the genus taxonomic level, a total of 54 genera were found to contribute to sulfur metabolism. The most abundant genus was *Treponema*, which harbored 80 genes (accounting for 32.4% of classified genes), followed by *Lactococcus* (27 genes), *Pseudomonas* (21 genes), *Clostridium* (15 genes), *Enterobacter* (12 genes), and *Desulfovibrio* (7 genes). However, *Treponema* and *Clostridim* were found to participate in cysteine/acetate production and assimilatory sulfate reduction only. Meanwhile, *Pseudomonas*, *Enterobacter*, and *Desulfovibrio* carried all sets of genes involved in all three pathway modules in sulfur metabolism. *Lactococcus* only possessed genes encoding enzymes for cysteine/acetate production (Table 2).

At the species level, 58 species were found to possess genes for sulfur metabolism. Of note, *Candidatus Azobacteroides pseudotrichonymphae*, *Delftia acidovorans*, *Dysgonomonas mossii*, *Pseudomonas fluorescens*, *Salmonella enterica*, *Stenotrophomonas maltophilia*, *Treponema azotonutricium*, *Treponema phagedenis*, and *Treponema primitia* were determined to have strong sulfur metabolizers, whereas *D. mossii*, *S. enterica*, and *S. maltophilia* were found to be involved only in dissimilatory sulfate reduction.

### 3.8. The Role of Freely Living Gut Prokaryotes in Nitrogen Recycling

**Figure 6:**
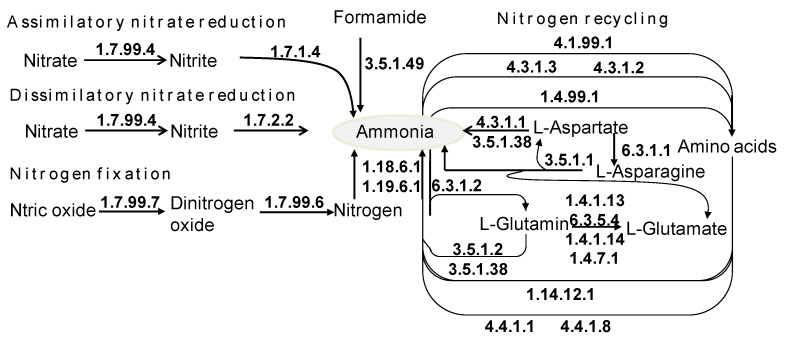
The KEGG-annotated enzymes involved in nitrogen metabolism that were mined from metagenomic DNA data of freely living prokaryotic community in *C. gestroi* gut.

Nitrogen metabolism and recycling plays a crucial role in the termite gut and is carried out by symbiotic bacteria that conserve nitrogen from a poorly nitrogenous diet, typically in wood. Nitrogen metabolism includes nitrogen reduction and fixation, and it is divided into six pathway modules: nitrogen fixation, assimilatory nitrate reduction, dissimilatory nitrate reduction, detrification, nitrification and complete nitrification, and comammox. Besides the six modules, nitrogen is also conserved in the form of nitrogenous compounds, representatively amino acids. From the metagenomic DNA data of free-living bacteria in the *C. gestroi* gut, we found a total of 709 genes encoding for 30 enzymes involved in assimilatory nitrate reduction, dissimilatory nitrate reduction, nitrogen fixation, glutamate metabolism, and amine metabolism (Table S9 and Figure 6). The most abundant genes were those contributing to glutamate metabolism, followed by the genes associated with nitrogen fixation and nitrate reduction (Table S9). The genes correlated to ammonia production through reducing nitrate were *nap*AGHIJVWYZ encoding for nitrate reductase (EC 1.7.99.4), *nrf*A encoding for formate-dependent nitrite reductase (EC 1.7.2.2) and *nir*BD encoding for nitrite reductase (EC 1.7.1.4), which convert nitrate into ammonia (Figure 6). Dinitrogen-fixing prokaryotes in the termite gut fix nitrogen in three ways: molybdenum-dependent (Nif), vanadium-dependent (Vnf), and iron-only alternative nitrogenases (Anf) [7,40]. We found accumulated genes *nif*DHK (105 genes) encoding for nitrogenase molybdenum–iron protein (EC 1.18.6.1) and a gene encoding for nitrogenase (EC 1.19.6.1) correlated to nitrogen fixation into ammonia via the molybdenum-dependent pathway only.

Diazotrophs situated freely in the *C. gestroi* gut and responsible for nitrogen fixation comprised both archaea (5 genes) and bacteria (704 genes). The archaeal genes related to nitrogen fixation, glutamate metabolism, and amine metabolism were found. There were 10 bacterial phyla harboring genes that took part in nitrogen metabolism, while Firmicutes was the most abundant, possessing 182 genes, followed by *Spirochaetes* (161 genes), *Proteobacteria* (152 genes), *Bacteroidetes* (90 genes), and *Synergistetes* (31 genes). The total genes from the five most abundant phyla accounted for 96.6% of the genes for nitrogen metabolism. The genes for nitrogen fixation appeared in all abundant phyla; however, only *Proteobacteria* bore the genes for assimilatory/dissimilatory nitrate reduction and nitrogen fixation (Table S9).

At the genus level, *Treponema* possessed the most genes related to nitrogen metabolism (132 genes), followed by *Lactococcus* (45 genes) and *Pseudomonas* (39 genes). At the species level, 299 genes related to nitrogen metabolism were classified into 80 species. The five most abundant species were *T. primitia* (70 genes), *T. azotonutricium*(32 genes), *L. raffinolactis*(27 genes), *C. Azobacteroides* pseudotrichonymphae (13 genes), and *D. gadei*(12 genes). Of the 73 classified genes for nitrogen fixation, *T. primitia* contributed 29 genes and *T. azotonutricium* contributed 15 genes. This clarifies the important role of *Treponema* in nitrogen metabolism in the termite gut.

### 3.9. The Role of Freely Living Gut Prokaryotes in Antibiotic Synthesis

**Figure 7:**
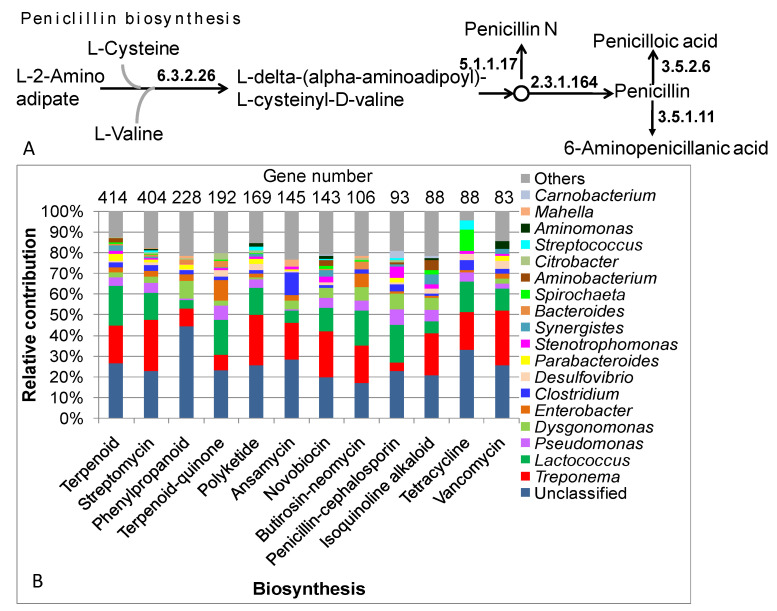
The role of free-living bacterial genera in *C. gestroi* gut in the biosynthesis of major antibiotics. (**A**) Penicillin biosynthesis pathway of the bacteria in the termite gut; (**B**) contribution of the main bacterial genera harboring genes coding for proteins/enzymes participating in main antibiotics’ biosynthesis.

From the metagenomic DNA data of the free-living prokaryotes of the *C. gestroi* gut, 2223 genes coding for 97 enzymes/proteins involved in the biosynthesis of 17 antibiotic groups were mined, including vancomycin, isoquinoline alkaloid, tetracycline, penicillin and cephalosporin, ansamycin, novobiocin, polyketide, ubiquinone and other terpenoid-quinones, a terpenoid backbone, streptomycin, flavonoids, phenylpropanoids, butirosin and neomycin, stilbenoids, diarylheptanoids and gingerol, flavones and flavonols, betalain, and 12-, 14-, and 16-membered macrolides (Table S10). The number of genes related to the biosynthesis of flavonoids, stilbenoids, diarylheptanoids and gingerol, flavones and flavonols, betalain, and 12-, 14-, and 16-membered macrolides was limited. Meanwhile, the genes for the synthesis of terpenoid backbones and streptomycin were predominant, accounting for 36.8% of the total genes for antibiotic synthesis. In general, the pathways for antibiotic biosynthesis are complicated; thus, even though large numbers of genes were mined, only the pathway for penicillin biosynthesis by bacteria in the *C. gestroi* gut was found to be relatively complete. In the first step, delta-(L-alpha-aminoadipyl)-L-cysteinyl-D-valine synthetase (EC 6.3.2.26) condenses three leading amino acids, L-2-aminoadipate, L-cysteine, and L-valine, into L-delta-(alpha-aminoadipoyl)-L-cysteinyl-D-valine, a so-called tripeptide. In theory, the tripeptide is oxidized and, under the catalytic activity of isopenicillin N synthase (EC 1.21.3.1), isopenicillin N is synthesized. However this enzyme was not found in the data. Isopenicillin N is a weak antibiotic that could be converted into penicillin N by isopenicillin-N epimerase (EC 5.1.1.17) or penicillin by isopenicillin-N N-acyltransferase (EC 2.3.1.164). The penicillin in the termite gut can be degraded by beta-lactamase (EC 3.5.2.6) or penicillin amidase (EC 3.5.1.11) (Figure 7A).

Of the 2223 genes found to participate in antibiotic synthesis, 1480 genes were classified into 16 bacterial phyla, 143 genera, and 176 species. The genera *Treponema, Lactococcus, Pseudomonas, Dysgonomonas, Enterobacter,* and *Clostridium* were the six most dominant and accounted for ~50% of the genes involved in all 17 antibiotic groups. *Treponema* was the most abundant genus, with 401 genes, supporting 10–20% of the total genes for the synthesis of terpenoids, streptomycin, polyketide, ansamycin, novobicin, butirosin-neomycin, isoquinoline alkaloids, tetracycline, and vancomycin. However, *Treponema* possessed fewer genes involved in the synthesis of terpenoid-quinones, phenylpropanoids, and penicillin-cephalosporin. *T. azotonutricium* was the most dominant and harbored genes involved in the synthesis of many antibiotics (Figure S4). *Lactococcus* was the second most abundant genus, harboring a large proportion of genes (281 genes) for the synthesis of nine antibiotics, especially those involved in penicillin-cephalosporin synthesis. In *Enterobacter*, the genes for terpenoid-quinone synthesis dominated over the genes for the other antibiotics. While *Clostridium* was found to possess many genes for the production of ansamycin, *Desulfovibrio* harbored the majority of genes for streptomycin and terpenoid-quinone synthesis (Figure 7B). For the production of penicillin, except for unclassified bacteria, *Lactococcus* contributed 18.3% of genes. At the species level, *L. raffinolactis*, *L. lactis*, and *L. garvieae* play important roles in the production of many antibiotics. The genes from *Lactococcus* species accounted for a maximum ~40% of the genes producing terpenoids (Figure S4).

### 3.10. The Role of Freely Living Gut Prokaryotes in Chemical Degradation

Termites are regarded as “soil engineers”, enhancing the physical and chemical properties of soils and contributing nutrients and minerals to adjacent soil. The soils from termite mounds are preferred for agriculture to grow vegetables and crops in many places all over the world [41]. Consistently, besides the pathways related to normal catabolism, such as the catabolism of amino acids, RNA, DNA, purin, pyrimidin, sugars, polysaccharide degradation, etc., we also observed enzymes involved in 18 pathways for the degradation of aromatic compounds and chemicals (Table 3). All 18 chemical compounds, including benzoate, benzoate-linked chemicals, methylnaphthalene, naphthalene and anthracene, tetrachloroethene, 3-chloroacrylic acid, hexachlorocyclohexane, geraniol, trinitrotoluene, atrazine, caprolactam, styrene, 2,4-dichlorobenzoate, toluene, xylene, 1,4-dichlorobenzene, 1,2-dichloroethane, fluorobenzoate, and carbazole, display negative impacts on human health and contaminate the soil.

Of these 18 pathways, the pathways for the degradation of benzoate and benzoate compounds, such as benzoate degradation via CoA ligation, benzoate degradation via hydroxylation, and 2,4-dichloroben-zoate degradation, were dominant in this study. Of the aromatic compounds, geraniol, atrazine, styrene and 1,4-dichlorobenzene are present in the pesticides used against termites for the preservation of wooden constructions. The eight pathways for the degradation of methylnaphthalene, naphthalene and anthracene, tetrachloroethene, 3-chloroacrylic acid, 2,4-dichlorobenzoate, toluene and xylene, 1,2-dichloroethane, fluorobenzoate, and carbazole were first observed in the termite gut. The enzymes participating in important steps for the conversion of aromatic compounds, including 4-hydroxy-4-methyl-2-oxoglutarate aldolase (EC 4.1.3.17), acetyl-CoA acyltransferase (EC 2.3.1.16), 3-oxoadipyl-CoA thiolase (EC 2.3.1.174), acetyl-CoA C-acetyltransferase (EC 2.3.1.9), salicylate hydroxylase (EC 1.14.13.1), 1,6-dihydroxycyclohexa-2,4-diene-1-carboxylate dehydrogenase (EC 1.3.1.25), aldehyde dehydrogenase (NAD+)(EC 1.2.1.3), carboxymethylenebutenolidase (EC 3.1.1.45), 2-haloacid dehalogenase (EC 3.8.1.2), acetyl-CoA C-acetyltransferase (EC 2.3.1.16), hydrogenase (1.2.7.1), pyruvate synthase (EC 1.12.99.6), urease subunit alpha (EC 3.5.1.5), allophanate hydrolase (EC 3.5.1.54), 3-hydroxyacyl-CoA dehydrogenase (EC 1.1.1.35), propionate CoA-transferase (EC 2.8.3.1), fumarylacetoacetase (EC 3.7.1.2), 4-hydroxy-2-oxovalerate aldolase (EC 4.1.3.39), benzaldehyde dehydrogenase (NAD)(EC 1.2.1.28), haloacetate dehalogenase (EC 3.8.1.3), and carboxymethylenebutenolidase (EC 3.1.1.45), are indicated in Table 3. Of the 1789 genes related to the 18 degradation pathways, 1624 genes were categorized into 18 phyla, 1351 genes were distributed into 172 genera, and 875 genes originated from 212 species. The four most abundant phyla, harboring 91.5% of genes, were Proteobacteria (546 genes), Firmicutes (499 genes), and Spirochaetes (267 genes), followed by Bacteroidetes (174 genes). At the genus level, the maximum abundance was found for *Treponema* (232 genes, accounting for 17.2%), followed by *Pseudomonas* (204 genes) and *Lactococcus* (170 genes). Species that contributed many genes for aromatic compound degradation were *T. primitia*(123 genes), *T. azotonutricium*(69 genes), *L. raffinolactis*(65 genes), *L. lactis* (49 genes), *L. garvieae*(41 genes), and *P. fluorescens* (41 genes), which were also the most abundant species in the termite gut.

## 4. Discussion

### 4.1. Diversity of Prokaryotic Community Living Freely in C. gestroi Gut Reveals Unique Attributes of the Dominant Types

Lower termites’ digestive tracts contain major proportions of flagellates (accounting for 90% of digestive volume), prokaryotic-associated flagellates (on the surface and inside of flagellates), and attaching and free-living prokaryotes (accounting for 7–10%) [2,3,4]. In the current study, the free-living prokaryotic community in the lower termite *C. gestroi*’s gut was analyzed based on metagenomic DNA sequencing to elucidate the role of the community for the host. Accordingly, 80% of the genes from the metagenomic DNA data belonged to bacteria and only a few genes (0.42%) were of archaeal origin. The dominant phyla were specified by the greatest abundance of Firmicutes (accounting for 29% of bacterial genes), followed by Proteobacteria (23%), Spirochaetales (23%), Bacteroidetes (15%), and Synergistetes (6%) (Table S2). Fibrobacteres were less abundant, accounting for 0.02%. In agreement with these results, Firmicutes, Bacteroidetes, and Spirochaetes were the three most dominant phyla in the guts of wood-feeding termites, which are also major decomposers of cellulose and hemicellulose in higher termites. In addition, Fibrobacteres is rare in lower termites but is a significant phylum in non-Macrotermitinae wood-feeding Termitidae [7]. Earlier, an analysis of the 16S rDNA clonal library in the lower termite *Reticulitermes lucifugus*’s gut also indicated that Firmicutes, Proteobacteria, Spirochetes, and Bacteroidetes were the most abundant phyla and Candidatus TG1 also was among the four most abundant phyla [42]. However, TG1 is specific to flagellate-associated bacteria, which were absent in this report. The dominant free-living bacteria in the *C. gestroi* gut were different from the dominant ones in other lower termite guts. The analysis of 16S rDNA regions V3–V4 of the total bacteria in the guts of two higher termites and two lower termites, *Tsaitermes ampliceps* and *Reticulitermes flaviceps*,demonstrated that Bacteroidetes was the most abundant in *T. ampliceps* and *R. flaviceps* [43]. In contrast, Firmicutes was the most abundant phylum freely living in the gut of lower termite *C. gestroi*. In higher termites, Spirochaetes was the most dominant [43], but in lower termites, including *C. gestroi*, *Synergistetes* accounted for about 5% of the bacterial genes.

The structure of the dominant free-living bacteria in the *C. gestroi* gut was also quite different from the structures in lower termites such as *T. ampliceps* and *R. flaviceps* [43]. Accordingly, the ratio of Firmicutes:Proteobacteria:Spirochaetes:Bacteroidetes: Synergistetes in bacterial symbionts from the *C. gestroi* gut was 5.2:4.1:4.0:2.7:1 (Table S2), while the ratio in the bacterial community from the *T. ampliceps* and *R. flaviceps* gut was approximately 3.6:2.8:2.2:6:1 [43]. The overall picture of the diversity suggested that the structure of the abundant phyla living freely in the *C. gestroi* gut resembled that of the soil-feeding termites and was substantially different from other groups of termites, including lower termites, non-Macrotermitinae wood-feeding Termitidae, and fungal-cultivating termites described by Arora et al. (2022) (Figure S5). The result partly reflects that the bacterial community living freely in the gut of lower termite *C. gestroi* is quite different from that in the guts of lower termites like *T. ampliceps* and *R. flaviceps*.

The ratio of Firmicutes versus Bacteroidetes freely living in the termite gut was rather high. Bacteroidetes play an important role in the digestion of complex lignocellulose, with many branches composed of different types of 5C sugars that extend from the xylan backbone. Firmicutes are responsible for cellulose digestion and are considered as the main lignocellulose degraders due to harboring genes encoding diverse CAZymes including GHs, GTs, CEs, CBMs, and AAs. Meanwhile, Bacteroidetes seem to be adapted to digest complex hemicellulose components due to the possession of a high proportion (42.1%) of CBMs and only a few GHs (8.4%), as was seen in the previous study on rumens’ microbial diversity [44,45,46]. The low ratio of Firmicutes versus Bacterioidetes is used as an indicator for effective lignocellulose degradation on an industrial scale [47]. In this study, the ratio was quite high as compared to other studies (Table 4), similar to that of grass-feeding termite *C. cumulans.* The lowest ratio has been reported in the cockroach gut and the highest ratio was reported in non-Macrotermitinae wood-feeding Termitidae (Table 4). Primarily, termites with more than 3000 described species are demonstrated to feed on wood. The wood digestion in termite guts is based on both termites’ and guts’ microbial metabolism, depending upon the termite lineage and diet. Bacteria constitute only one part of the microbial community in the termite gut, besides protozoa (in lower termites) and fungi (in higher termites). Additionally, different methodologies are generally applied for the investigation of the bacterial community composition in termite guts, as has been reported in various studies. Thus, there are multiple reasons that support the varying ratios of Firmicutes/Bacterioidetes in the termite gut. Spirochaetes are dominant in wood-feeding termites, accompanied by Fibrobacteres and/or Firmicutes, which are the major decomposers of cellulose and hemicellulose in higher termites [48,49,50]. Bacteroidetes are more abundant in the lower termite gut, but have low abundance in non-Macrotermitinae Termitidae. Fibrobacteres are rare in lower termites but are a significant phylum in non-Macrotermitinae wood-feeding Termitidae [7]. Thus, Spirochaetes, Fibrobacteres, Firmicutes, and Bacteroidetes are the most important phyla in the termite gut for lignocellulose degradation. In free-living bacterial symbionts in the *C. gestroi* gut, Spirochaetes, Fibrobacteres, Firmicutes, and Bacteroidetes have been reported to occupy 67.8% of the total genes. Accordingly, applying 40 markers for the analysis of the taxonomic distribution of the major bacterial groups in 74 termite lineages belonging to four termite groups showed that four phyla, Spirochaetes, Fibrobacteres, Firmicutes, and Bacteroidetes, occupied 65.3%, 68.0%, and 70.9% of the bacterial genes in the guts of soil-feeding termites (SF), fungal-cultivating termites (FC), and lower termites (LT), respectively. Meanwhile, in the case of non-Macrotermitinae wood-feeding Termitidae, these major phyla in the gut reached up 95.0% of the total bacterial genes (Figure S5) [7]. In agreement with our result, Fibrobacteres has been reported to be less abundant in lower termites and was not seen in the guts of *Coptotermes curvignathus* and *C. formosanus* [51,52]. In general, the proportion of the four phyla is higher than 65% in the guts of all higher and lower termites (Table 4), indicating the role of these bacteria in the termite gut. Taking all the above into account, the ratio of the dominant phyla Firmicutes/Bacteroidetes in the bacterial community living freely in the *C. gestroi* gut was similar to that observed previously in grass-feeding higher termite *Cornitermes cumulans*, and the richness of the six most abundant phyla was the closest to that in fungal-cultivating termites.

In this study, archaea represented only 0.5% of the free-living prokaryotes in the gut of *C. gestroi*. In the species taxonomy, the six most abundant species, including *M. smithii, M. ruminantium, M. hungatei, M. acetivorans, M. fervidus,* and *M. liminatans*, all belonged to the Euryarcheaota phylum (Figure S2). Consistent with our findings, another study reported that archaea occupied less than 1% of the prokaryotes in wood-feeding termite guts, while the proportion was much higher in *Macrotermitinae*(up to 4.6%) and even up to 10.6% in the soil-feeding termite *Mimeutermes* [7]. Methanogens usually constitute the majority in archaeal cells in the termite gut but are less diverse [56]. To the best of our knowledge, these abundant archaeal species were first reported from lower termite guts but have been isolated in different sources. For example, *M. smithii*is usually seen in the human gut and in contaminated water [57] and *M. ruminantium* predominates in ruminant livestock species [58], whereas *M. hungatei* and *Methanosarcina acetivorans* have been isolated from soil and thoroughly studied [59,60].

### 4.2. Contribution of CAZymes of Free-Living Prokaryotes of C. gestroi Gut in Lignocellulose Digestion

The termite gut is regarded as an effective lignocellulose-decomposing bioreactor. In the current study, we found 2175 bacterial domains of CAZymes/2165 genes encoding for 1300 GHs, 554 GTs, 26 PLs, 172 CEs, 22 AAs, and 101 CBMs (Table 1). Almost all of the CBMs found in this study are capable of binding to cellulose and hemicellulose and some CBMs have the potential to bind to chitin and pectin (Table S4). The AA family in this study was found to be responsible for the conversion of a wide range of phenolic compounds and lignocellulose, including oxidative cleavage based upon the activity of copper-dependent lytic polysaccharide monooxygenases (LPMOs). Compared to the CAZyme profile of 129 termite species belonging to lower termites, non-Macrotermitinae wood-feeding Termitidae, soil-feeding termites, and fungal-cultivating termites, a total of 50 CAZyme domains, including 3 families of AAs, 14 families of CBMs, CE3, CE6, 18 subfamilies of GHs, 5 families of GTs, and 7 subfamilies of PLs were found in the metagenomic DNA data, not previously reported in lower termite guts, in a study by Arora et al. (2022). In contrast, AA1, CBM23, CBM36, CBM4, CBM42, CBM57, CBM61, CBM63, CE12, CE13, CE16, GH101, GH104, GH112, GH114, GH117, GH121, GH126, GH138, GH14, GH33, GH44, GH45, GH46, GH47, GH48, GH49, GH52, GH54, GH59, GH6, GH62, GH64, GH70, GH72, GH79, GH81, GH84, GH87, and GH98 were not observed in this study [7]. Arora et al. (2022) showed that seven GHs, including GH44, GH45, GH5_1, GH5_38, GH5_46, GH8, and GH9, with cellulase activity were significantly depleted in lower termites [7] and were also less abundant in the guts of *C. gestroi*. Even GH44, GH45, GH5_1, and GH5_38 were absent in the free-living bacteria in the *C. gestroi* gut. The combined 65 GH subfamilies identified for cellulase and hemicellulase activities accounted for 28.5% of the total CAZymes. This proportion was similar to the enzymes from fungal-cultivating termites (28.5%) and higher than the enzymes from lower termite groups (25.4%) but lower than for oil-feeding termites (35.4%) and non-Macrotermitinae wood-feeding Termitidae (39.8%). These results were supported by a previous analysis indicating similar proportions of the six most abundant phyla in fungal-cultivating termites. However, the proportion of the cellulases and hemicellulases per GH family reached 47.6% in the gut of *C. gestroi*, higher than the proportion in lower termites (43.5%) and fungal-cultivating termites (44.5%) and lower than that from soil-feeding termites (55.1%) and non-Macrotermitinae wood-feeding Termitidae (56.7%) [7]. In a heatmap analysis of the relative abundance of CAZymes in this study and that in four groups of termites and cockroaches as described by Arora (2022), we found that CAZymes from free-living bacteria in the *C. gestroi* gut do not have a close relationship with any group of corresponding enzymes from a certain termite group (Figure S6). The GH family from *C. gestroi* was in a cluster of enzymes from fungal-cultivating termites, cockroaches, and lower termites, but CBMs from *C. gestroi* were more abundant than in other termite groups and, thus, were situated in a distinct branch in the phylogeny heatmap (Figure S6).

### 4.3. Free-Living Prokaryotes of C. gestroi Gut Interact with Each Other to Exhibit Diverse Nutritional Functions and Provide Health Benefits to Their Hosts

Termites are regarded as efficient lignocellulose degraders with a cellulose degradation rate of about 74–99% and hemicellulose conversion rate of 65–87%, producing carbon dioxide (42% carbon from wood), termite tissues (18% carbon from wood) [23], and a lot of hydrogen molecules (two liters of hydrogen from one paper sheet) [21]. In this study, the released hydrogen and carbon dioxide entered three prokaryotic pathways for acetate production as an important energy source for the host: (1) acetogenesis by acetogens, fixing CO_2_ and reducing H_2_ to synthesize acetate by the Wood–Ljungdahl pathway; (2) methanogenesis by archaeal methanogens, synthesizing methane from H_2_/CO_2_, followed by the action of methanotrophs (both archaea and bacteria) to oxidize the released methane into D-fructose-6P to limit the emission of methane into the atmosphere; (3) sulfur metabolism by sulfur-reducing bacteria to recycle sulfur and carbon during the final step of the Wood–Ljungdahl pathway for the efficient production of acetate. Free-living bacteria in the termite gut also play an essential role in nitrogen fixation to conserve nitrogen from the poorly nitrogenous diet in wood. Genetically, the Wood–Ljungdahl pathway of free-living bacteria in the termite gut was similar to that found in whole bacteria in other termites’ guts in a previous study [39], with the strong reduction of carbon dioxide to formate by formate dehydorgenase encoded by *fdhf* genes (Figure 4). Consistent with the previous study [7], *fdhF* and *acs* in this study were simultaneously presented with the greatest number of genes related to the WLP and distributed in acetogenic species. Although no species covered all enzymes for the WLP, the most significant numbers of genes coding for WLP enzymes were predicted to come from *T. primitia* and *L. raffinolactis.* Based on the ratio of total WLP enzymes versus total steps in the WLP, *L. lactis, C. Azobacteroides* pseudotrichonymphae*, D. gadei, L. garvieae,* and *S. caldaria* were identified to be the best potential candidates. To date, *T.primitia* [61], *Candidatus Treponema* intracellularis [62], and *Candidatus Adiutrix* [63] have been speculated to be responsible for carrying out reductive acetogenesis in the termite gut. Thus, besides the known acetogens, *L. lactis, L. garvieae, L. raffinolactis, D. gadei,* and *C. Azobacteroides* pseudotrichonymphae were shown to be important acetogenics in the *C. gestroi* gut for the first time. The three most abundant phyla containing acetogens in the *C. gestroi* gut are also found as acetogenic sources in different environments [24], including the intestinal tracts of animals [64], humans [65], and termites, as well in soils and water.

Normally, sulfur metabolism is divided into four pathway modules: assimilatory sulfate reduction, disssimilatory sulfate reduction, thiosulfate oxidation by SOX, and cysteine biosynthesis. However, for the first time, we reported only three modules in the freely living prokaryotic community of *C. gestroi* (Figure 5), while bacteria prohibited cysteine/acetate biosynthesis. Although *Treponema* was the most abundant sulfate-reducing bacterium, *Pseudomonas, Enterobacte,* and *Desulfovibrio* were the best potential sulfate reducers because of the presence of genes participating in all three pathway modules (Table 2). *Desulfovibrio* has been found to be a common genus generating energy via sulfate respiration in the termite gut and even in the human gut [66,67]. Earlier, in an analysis of the gut metagenomic DNA of 10 lower termites, Arora et al. indicated that four dominant phyla, including Planctomycetes, Desulfobacteria, Actinobacteria, and Firmicutes, play an important role in sulfur metabolism in lower termites. However, only *Desulfovibrio* and *Pilibacter*are assigned this function [7]. In this study, diverse bacterial genera were found to possess genes for sulfur metabolism. For example, in addition to *Desulfovibrio*, *Pseudomonas* and *Enterobacter* also played an important role in sulfate respiration.

In addition, for the first time, only four out of seven known pathway modules for nitrogen metabolism and recycling were observed in the free-living bacteria in the gut of *C. gestroi*. These modules were assimilatory nitrate reduction, dissimilatory nitrate reduction, nitrogen fixation, and nitrogen recycling (including glutamate metabolism and amine metabolism), while they lacked three pathway modules, namely denitrification, nitrification and complete nitrification, and comammox.

The main outcome of this research work was the identification of 18 possible pathways for the degradation of aromatic compounds and chemicals (Table 3). For the degradation of benzoate and benzoate compounds, CoA ligation and hydroxylation and 2,4-dichloroben-zoate degradation were found to be the dominant pathways in this study, and the same has also been observed in both wood-feeding termite guts [68] and termite mound soils [41]. However, the degradation of fluorobenzoate seen in our metagenomic data has not been reported in the two previous studies. The degradation of hexachlorocyclohexane, geraniol, trinitrotoluene, atrazine, caprolactam, styrene, hexachlorocyclohexane, and 1,4-dichlorobenzene reported here has also been observed in various individual studies on termite guts. Interestingly, many compounds, such as geraniol, atrazine, styrene and 1,4-dichlorobenzene, are components of the pesticides used against termites for the preservation of wooden constructions. In this study, the termites were harvested from nests in wooden pagodas and furniture in Ha Noi, Vietnam. This result reveals that the bacteria in the termite gut have adapted to decompose these types of pesticides in order to assist their hosts in surviving via the partial neutralization or deactivation of such toxin compounds. This evidence is supported by the fact that gut microbial metagenomes are the second host genomes, which play a vital role in the host’s optimal growth and development.

Many pathways were observed in this study for the first time in the termite gut, including the pathways for the degradation of methylnaphthalene, naphthalene and anthracene, tetrachloroethene, 3-chloroacrylic acid, 2,4-dichlorobenzoate, toluene and xylene, 1,2-dichloroethane, fluorobenzoate, and carbazole.

The free-living prokaryotes in the termite gut not only supply nutrients and energy and carry out the detoxification of harmful chemicals, but also produce antibiotics that may protect the host from pathogenic agents. In this metagenomic DNA study, 2223 genes coding for 97 enzymes/proteins involved in the biosynthesis of 17 antibiotic groups, including vancomycin, isoquinoline alkaloids, tetracycline, penicillin and cephalosporin, ansamycin, novobiocin, polyketide, ubiquinone and other terpenoid-quinones, terpenoid backbones, streptomycin, flavonoids, phenylpropanoids, butirosin and neomycin, stilbenoids, diarylheptanoids and gingerol, flavones and flavonols, betalain, and 12-, 14-, and 16-membered macrolides (Table S10), were mined. Although no complete antibiotic biosynthesis pathway was found, the valuable insights and preliminary findings of this study could pave the way for the isolation of new antibiotic-producing bacteria from the termite gut.

## 5. Conclusions

The present study sheds light on the role of the free-living prokaryotic community in the *C. gestroi* gut. With the typical structure and diversity of the microbiota, the prokaryotic community provides enzymes participating in lignocellulose degradation to generate sugars for the host; produces acetate to supply energy for the host; recycles carbon, nitrogen, and sulfur to contribute nutrients for the host; detoxifies toxic aromatic compounds to ensure the survival and good health of termites; and harbors many genes for antibiotic synthesis to possibly protect the host from pathogenic agents. Besides providing benefits to their host termites, the gut microbes can potentially act as new primary biological resources to enhance lignocellulose conversion in biorefineries; increase the digestion of lignocellulose in feedstuffs for improved animal husbandry; aid in the production of biofertilizers for agricultural purposes; aid in the discovery and production of newer antibiotics; and finally enable applications in the bioremediation of recalcitrant environmental pollutants.

## Figures and Tables

**Table 1:** List of domains of carbohydrate-active enzymes that were mined by dbCAN2 and CAZy databases.

CAZy Family	Domain Number	%	CAZy Family	Gene Number	%	CAZy Family	Gene Number	%	CAZy Family	Gene Number	%
**AA:**	**22/22 genes**	**1.011**	GH3	90	4.14	GH115	5	0.23	**GT: 554/554 genes**		**25.471**
AA4	7	0.32	GH1	77	3.54	GH137	5	0.23	GT2	170	7.82
AA10	6	0.28	GH29	67	3.08	GH141	5	0.23	GT4	92	4.23
AA3	5	0.23	GH109	52	2.39	GH142	5	0.23	GT51	73	3.36
AA6	2	0.09	GH23	51	2.34	GH144	5	0.23	GT5	35	1.61
AA2	1	0.05	GH78	48	2.21	GH37	5	0.23	GT35	27	1.24
AA7	1	0.05	GH92	35	1.61	GH76	5	0.23	GT28	20	0.92
**CBM:**	**101/95 genes**	**4.644**	GH73	30	1.38	GH102	4	0.18	GT83	20	0.92
CBM67	25	1.15	GH95	30	1.38	GH108	4	0.18	GT19	16	0.74
CBM32	16	0.74	GH77	29	1.33	GH113	4	0.18	GT9	16	0.74
CBM48	11	0.51	GH106	25	1.15	GH133	4	0.18	GT26	12	0.55
CBM62	8	0.37	GH2	24	1.10	GH136	4	0.18	GT1	10	0.46
CBM13	5	0.23	GH18	22	1.01	GH103	3	0.14	GT8	10	0.46
CBM34	6	0.28	GH20	21	0.97	GH120	3	0.14	GT30	8	0.37
CBM6	6	0.28	GH4	21	0.97	GH140	3	0.14	GT27	5	0.23
CBM51	5	0.23	GH5	20	0.92	GH19	3	0.14	GT81	5	0.23
CBM9	5	0.23	GH127	19	0.87	GH27	3	0.14	GT101	4	0.18
CBM20	3	0.14	GH51	19	0.87	GH63	3	0.14	GT21	4	0.18
CBM35	2	0.09	GH31	17	0.78	GH11	2	0.09	GT3	4	0.18
CBM41	2	0.09	GH28	16	0.74	GH128	2	0.09	GT14	3	0.14
CBM73	2	0.09	GH38	16	0.74	GH129	2	0.09	GT20	3	0.14
CBM22	1	0.05	GH42	16	0.74	GH139	2	0.09	GT32	3	0.14
CBM5	1	0.05	GH65	15	0.69	GH145	2	0.09	GT56	3	0.14
CBM50	1	0.05	GH10	14	0.64	GH24	2	0.09	GT11	2	0.09
CBM66	1	0.05	GH35	14	0.64	GH50	2	0.09	GT84	2	0.09
CBM77	1	0.05	GH130	13	0.60	GH55	2	0.09	GT10	1	0.05
**CE:**	**172/171 genes**	**7.908**	GH30	13	0.60	GH94	2	0.09	GT104	1	0.05
CE10	45	2.07	GH36	13	0.60	GH110	1	0.05	GT25	1	0.05
CE4	37	1.70	GH57	13	0.60	GH123	1	0.05	GT39	1	0.05
CE1	32	1.47	GH88	13	0.60	GH143	1	0.05	GT70	1	0.05
CE9	31	1.43	GH105	12	0.55	GH15	1	0.05	GT73	1	0.05
CE11	10	0.46	GH25	12	0.55	GH17	1	0.05	GT94	1	0.05
CE7	5	0.23	GH97	12	0.55	GH53	1	0.05	**PL: 26/24 genes**		**1.195**
CE15	3	0.14	GH125	10	0.46	GH66	1	0.05	PL1	6	0.28
CE3	3	0.14	GH32	10	0.46	GH68	1	0.05	PL22	5	0.23
CE14	2	0.09	GH9	10	0.46	GH74	1	0.05	PL8	4	0.18
CE8	2	0.09	GH39	8	0.37	GH85	1	0.05	PL9	3	0.14
CE2	1	0.05	GH116	7	0.32	GH89	1	0.05	PL11	2	0.09
CE6	1	0.05	GH8	7	0.32	GH91	1	0.05	PL17	2	0.09
**GH:**	**1300/1298 genes**	**59.770**	GH16	6	0.28	GH93	1	0.05	PL5	2	0.09
GH13	145	6.67	GH26	6	0.28	GH99	1	0.05	PL6	1	0.05
GH43	91	4.18	GH67	6	0.28				PL7	1	0.05

The bold numbers indicate total domains/total genes for six CAZyme families.

**Table 2:** The four most abundant free-living phyla in *C. gestroi* gut involved in sulfur metabolism.

Phylum	Genus	Species	Pathway Module	Gene Number
Spirochaetes				85
	*Treponema*			80
		*Treponema azotonutricium*	Assimilatory sulfate reduction	1
		*Treponema azotonutricium*	Cysteine/acetate biosynthesis	20
		*Treponema phagedenis*	Assimilatory sulfate reduction	2
		*Treponema phagedenis*	Cysteine/acetate biosynthesis	1
		*Treponema primitia*	Assimilatory sulfate reduction	7
		*Treponema primitia*	Cysteine/acetate biosynthesis	37
Proteobacteria				80
	*Pseudomonas*		Assimilatory sulfate reduction	5
	*Pseudomonas*		Cysteine/acetate biosynthesis	9
	*Pseudomonas*		Dissimilatory sulfate reduction	7
		*Pseudomonas fluorescens*	Assimilatory sulfate reduction	1
		*Pseudomonas fluorescens*	Cysteine/acetate biosynthesis	2
	*Enterobacter*		Assimilatory sulfate reduction	3
	*Enterobacter*		Cysteine/acetate biosynthesis	8
	*Enterobacter*		Dissimilatory sulfate reduction	1
	*Desulfovibrio*		Assimilatory sulfate reduction	4
	*Desulfovibrio*		Cysteine/acetate biosynthesis	2
	*Desulfovibrio*		Dissimilatory sulfate reduction	1
	*Stenotrophomonas*	*Stenotrophomonas maltophilia*	Cysteine/acetate biosynthesis	3
	*Stenotrophomonas*	*Stenotrophomonas maltophilia*	Dissimilatory sulfate reduction	1
	*Delftia*	*Delftia acidovorans*	Assimilatory sulfate reduction	1
	*Delftia*	*Delftia acidovorans*	Cysteine/acetate biosynthesis	2
	*Salmonella*	*Salmonella enterica*	Cysteine/acetate biosynthesis	1
	*Salmonella*	*Salmonella enterica*	Dissimilatory sulfate reduction	1
Firmicutes				72
	*Lactococcus*		Cysteine/acetate biosynthesis	27
	*Clostridium*		Assimilatory sulfate reduction	6
	*Clostridium*		Cysteine/acetate biosynthesis	9
Bacteroidetes			Assimilatory sulfate reduction	8
Bacteroidetes			Cysteine/acetate biosynthesis	22
Bacteroidetes			Dissimilatory sulfate reduction	7
	*Dysgonomonas*	*Dysgonomonas mossii*	Cysteine/acetate biosynthesis	1
	*Dysgonomonas*	*Dysgonomonas mossii*	Dissimilatory sulfate reduction	1
	*Candidatus Azobacteroides*	*Candidatus Azobacteroides pseudotrichonymphae*	Assimilatory sulfate reduction	4
	*Candidatus Azobacteroides*	*Candidatus Azobacteroides pseudotrichonymphae*	Cysteine/acetate biosynthesis	1

The shaded groups show the bacteria participating in all three pathway modules of sulfur metabolism.

**Table 3:** Pathways linked to the metabolism of aromatics in free-living bacteria in *C. gestroi* gut.

No.	Map	Degradation Pathway	Gene Number	Key Role of Important Enzymes	
				EC	
1	map00632	Benzoate degradation via CoA ligation	555	4.1.3.17	The last step to produce pyruvate, oxaloacetate, succinyl-CoA
				2.3.1.16, 2.3.1.174	The last step to produce pyruvate, oxaloacetate, succinyl-CoA
				2.3.1.9	The last step to generate succinyl-CoA
2	map00624	Methylnaphthalene degradation	502	1.14.13.1	The last step to generate acetyl-CoA
3	map00626	Naphthalene and anthracene degradation	323	1.14.13.1	The last step to generate catechol to enter the pathway for benzoate degradation or generate dihydroxynapthoate entering napthalene degradation pathway
4	map00625	Tetrachloroethene degradation	287	6.2.1-, 3.7.1-, 6.2.1-, 3.1.2.-, 1.1.-.-	The last step to generate catechol that can be further processed in benzoate degradation or generate methylcatechol to enter the pathway of xylene degradation
5	map00362	Benzoate degradation via hydroxylation	170	2.3.1.16, 2.3.1.174, 1.3.1.25	The last step to generate benzoyl-CoA
6	map00641	3-Chloroacrylic acid degradation	150	1.2.1.3	The last step to generate catechol
7	map00361	Hexachlorocyclohexane degradation	149	3.1.1.45, 3.8.1.2	The most important step to generate 3-chloroacrylic acid
8	map00281	Geraniol degradation	125	2.3.1.16	The last step to generate CO2, maleylacetate, glycolate
9	map00633	Trinitrotoluene degradation	110	1.2.7.1, 1.12.99.6	The last step to generate 3-methylcrolonyl-CoA
10	map00791	Atrazine degradation	80	3.5.1.5, 3.5.1.54	A step to generate 2,4-diamino 6 hydroxylaminotoluene
11	map00930	Caprolactam degradation	65	1.1.1.35	The last step to generate CO2
12	map00643	Styrene degradation	50	2.8.3.1	The last step to generate3-oxoadipyl-CoA to enter the pathway of benzoate degradation
				3.7.1.2	The last step to generate L-lactate
13	map00623	2,4-Dichlorobenzoate degradation	49	4.1.3.39	The last step to convert xylen into acetoacetate and fumarate
14	map00622	Toluene and xylene degradation	49	1.2.1.28, 4.1.3.39	The last step to generate pyruvate acetaldehyde
15	map00627	1,4-Dichlorobenzene degradation	35	3.1.1.45	An important step to convert toluenze into hydroxybenzoate and benzoate before entering the pathway of benzoate degradation
16	map00631	1,2-Dichloroethane degradation	23	3.8.1.2, 3.8.1.3	The last step to generate pyruvate or acetaldehyde
17	map00364	Fluorobenzoate degradation	22	3.1.1.45	An important step to generate maleylacetate before entering the pathway of benzoate degradation
18	map00629	Carbazole degradation	8	4.1.3.39	The last step to generate glycolate

**Table 4:** Comparison of dominant bacterial phyla ratios in termite gut.

Termite	Firmicutes/Bacteroi- detes	% Relative Abundance of Bacteroidetes, Spirochaetes, Firmicutes, and Fibrobacteres	Strategy Used to Study Bacterial Community	Ref.
Wood-feeding lower termite *Reticulitermes flaviceps*	1.3	78.9	Pyrosequencing of the 16S rRNA gene amplicons from gut	[43]
Wood-feeding lower termite *Tsaitermes ampliceps*	0.8	84.4		
Wood-feeding higher termite *Mironasutitermes shangchengensis*	1.0	75.9		
Fungus-feeding higher termite *Odontotermes formosanus*	0.6	73.7		
Subterranean lower termite *Reticulitermes virginicus*	0.6	81.8	V3 and V4 hyper-variable regions	[53]
Hardwood-feeding higher termite *Microcerotermes strunckii*	1	76.0	Deep sequencing of amplified 16S rRNA and ITS genes	[54]
Softwood-feeding higher termite *Nasutitermes corniger*	1	90.0		
Grass-feeding higher termite *Cornitermes cumulans*	**1.8**			
Oil/grass-feeding higher termite *Termes riograndensis*	2.5			
*Coptotermes gestroi*	**1.9**	**67.8**	Free-living bacteria in the gut, whole metagenome sequencing, diversity analysis based on alignment against NR database	**In this study**
Lower termite	0.2	**70.9**	Gut metagenomes of 74 termite samples belonging to 4 groups, analyzed based on 40 markers	[7]
Non-Macrotermitinae wood-feeding Termitidae	6.5	95.0		
Soil-feeding termites (SF)	1.4	**65.4**		
Fungal-cultivating termites (FC)	0.3	**68.0**		
Cockroach (CR)	0.0	83.2		
Lower termite	1.0	35.0	Gut metagenomes of 74 termite samples belonging to 4 groups, analyzed based on 16S rDNA amplicon	
Non-Macrotermitinae wood-feeding Termitidae	1.3	36.3		
Soil-feeding termites (SF)	1.3	35.1		
Fungal-cultivating termites (FC)	1.0	35.3		
Wood-feeding lower termite *Coptotermes curvignathus*	0.2	81.5	16S rDNA cloning and sequencing by Sanger	[51]
Wood-feeding lower termite *Coptotermes formosanus*	0.7	86.9	16S rDNA cloning and sequencing by Sanger	[52]
Mound-building higher termite *Cornitermes sp. (Co191)*	1.6	79.2	Three-compartment metagenomes of 6 termite gut samples analyzed by 16S rRNA V4 region	[55]
Soil-feeding higher termite *Cubitermes ugandensis (Cu122)*	2.0	75.2		
Higher termite*Microcerotermes parvus (Mp193)*	0.9	86.5		
Higher termite*Nasutitermes corniger (Nc150)*	3.1	78.5		
Higher termite *Neocapritermes taracua (Nt197)*	3.8	72.5		
Higher termite *Termes hospes (Th196)*	3.2	80.7		

The bold numbers indicate the ratios close to that in *C. gestroi* gut.

## Data Availability

The sequences of all 125,431 putative proteins deduced from 125,431 genes were detailed and made available in Table S1 in our previous publication [8]. The other data are available in Supplementary tables published with this article.

## Supplementary Materials

The following supporting information can be downloaded at: https://www.mdpi.com/article/10.3390/insects14110832/s1, Figure S1: Statistics of bacterial composition distribution freely living in *C. gestroi* gut; Figure S2. Statistics of archaeal composition distribution freely living in *C. gestroi* gut; Figure S3. The compositional contribution of bacteria harboring genes coding for CAZymes in the gut of *C. gestroi*; Figure S4. The compositional contribution of bacterial species harboring genes related to antibiotics’ synthesis in the gut of *C. gestroi*; Figure S5. Comparison of bacterial diversity at phylum level of bacteria freely living in *C. gestroi* gut in this study, with the average diversity of bacteria in 74 samples of termites (Arora et al., 2022); Figure S6. Relative abundance of CAZymes found in metagenomic DNA data of bacteria freely living in *C. gestroi* gut and bacteria in cockroach gut and 129 termite species belonging to four groups (Arora et al., 2022); Table S1. Statistics of the microorganism composition distribution freely living in *Coptotermes gestroi* gut; Table S2. The composition and diversity of free-living bacteria in the gut of termite *C. gestroi* harvested in Ha Noi, Vietnam; Table S3. The composition and diversity of free-living archaea in the gut of termite *C. gestroi* harvested in Ha Noi, Vietnam; Table S4. CAZy, dbCAN2 domain function, and origin of bacterial genes in the gut of termite *C. gestroi* harvested in Ha Noi, Vietnam; Table S5. Genes and origin of free-living prokaryotic genes coding enzymes participating in Wood–Ljungdahl pathway in the gut of termite *C. gestroi* harvested in Ha Noi, Vietnam; Table S6. Genes and origin of free-living prokaryotic genes coding enzymes participating in methanogenesis pathway in the gut of termite *C. gestroi* harvested in Ha Noi, Vietnam; Table S7. Genes and origin of the genes encoding enzymes participating in methane metabolism pathway in the gut of termite *C. gestroi* harvested in Ha Noi, Vietnam; Table S8. Genes and origin of the genes encoding enzymes participating in sulfur metabolism in the gut of termite *C. gestroi* harvested in Ha Noi, Vietnam; Table S9. Genes and origin of the genes encoding enzymes participating in nitrogen metabolism in the gut of termite *C. gestroi* harvested in Ha Noi, Vietnam; Table S10. Genes and origin of the genes encoding enzymes participating in antibiotic biosynthesis in the gut of termite *C. gestroi* harvested in Ha Noi, Vietnam.

## Abbreviations

KEGG: Kyoto Encyclopedia of Genes and Genomes; AGLuCu: alpha-glucuronidase; CAZy: Carbohydrate-Active enZYmes; CAZymes: carbohydrate-active enzymes; PAD: pro-oxidant, antioxidant, and detoxification enzymes; WLP: Wood–Ljungdahl pathway; LT: lower termite; SF: soil-feeding Termitidae, WF: wood-feeding Termitidae excluding Macrotermitinae; FC: fungus cultivating Macrotermitinae; RDP: Ribosomal Database Project; NR: non-redundant; ORF: open reading frame; GHs: glycoside hydrolases; GTs: glycosyl transferases; PL: polysaccharide lyases; CE: carbohydrate esterases; CBMs: carbohydrate-binding modules; AAs: auxiliary activity redox enzymes.
