# Supplementary material for: Understanding the Role of Free-Living Bacteria in the Gut of the Lower Termite Coptotermes gestroi Based on Metagenomic DNA Analysis
Source: Insects. 2023 Oct 24;14(11):832. doi: 10.3390/insects14110832 (PMC10671698; doi:10.3390/insects14110832)
Supplement: Supplementary file 1 [file insects-14-00832-s001.zip › 5 Suplementary Tables and figures 20230817.pdf]

Figure S1: Statistics of bacterial composition distribution free-living in *C. gestroi* guts

Total: 95751 genes

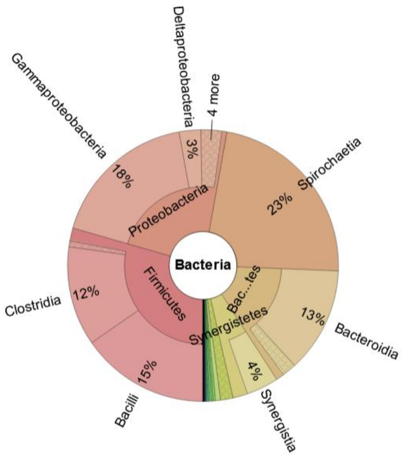

At class level

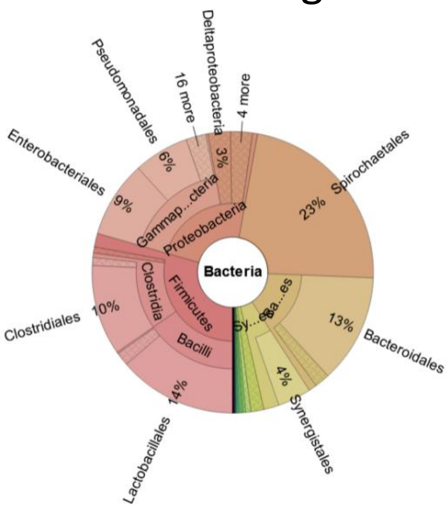

At order level

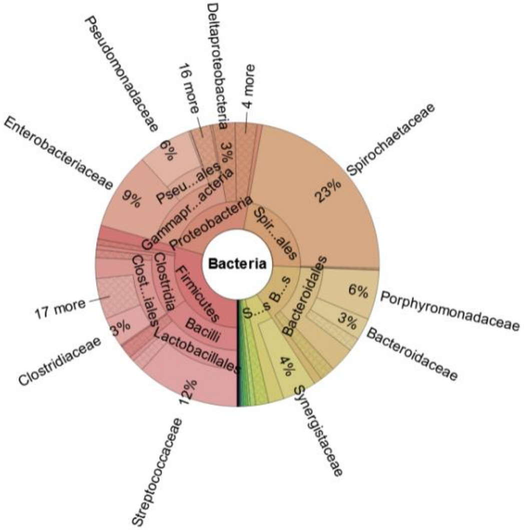

At family level

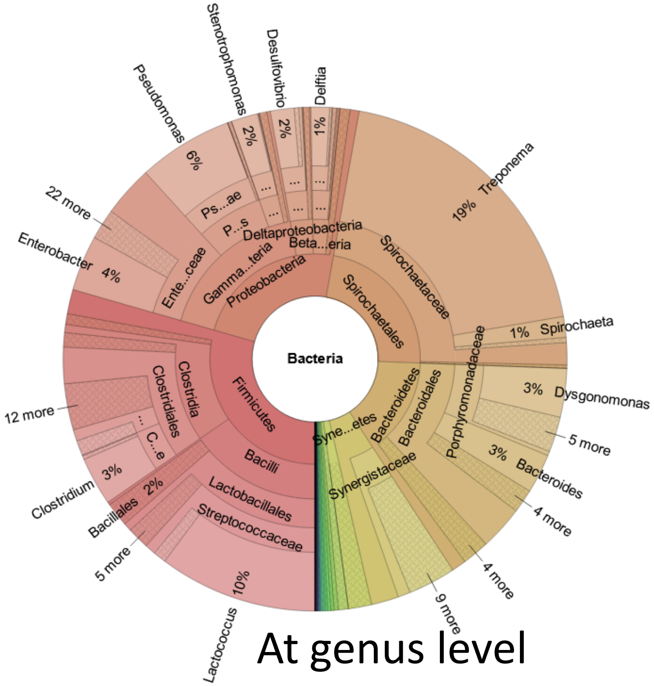

At genus level

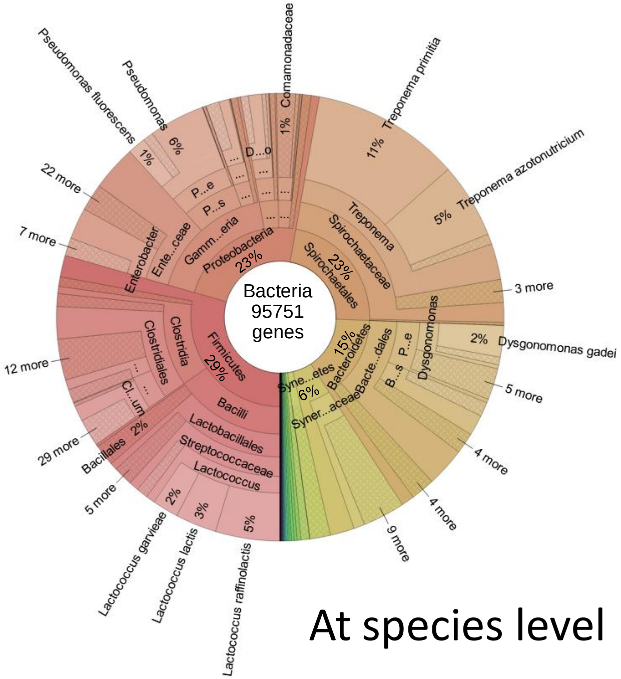

At species level

Figure S2. Statistics of archaeal composition distribution free-living in *C. gestroi* guts

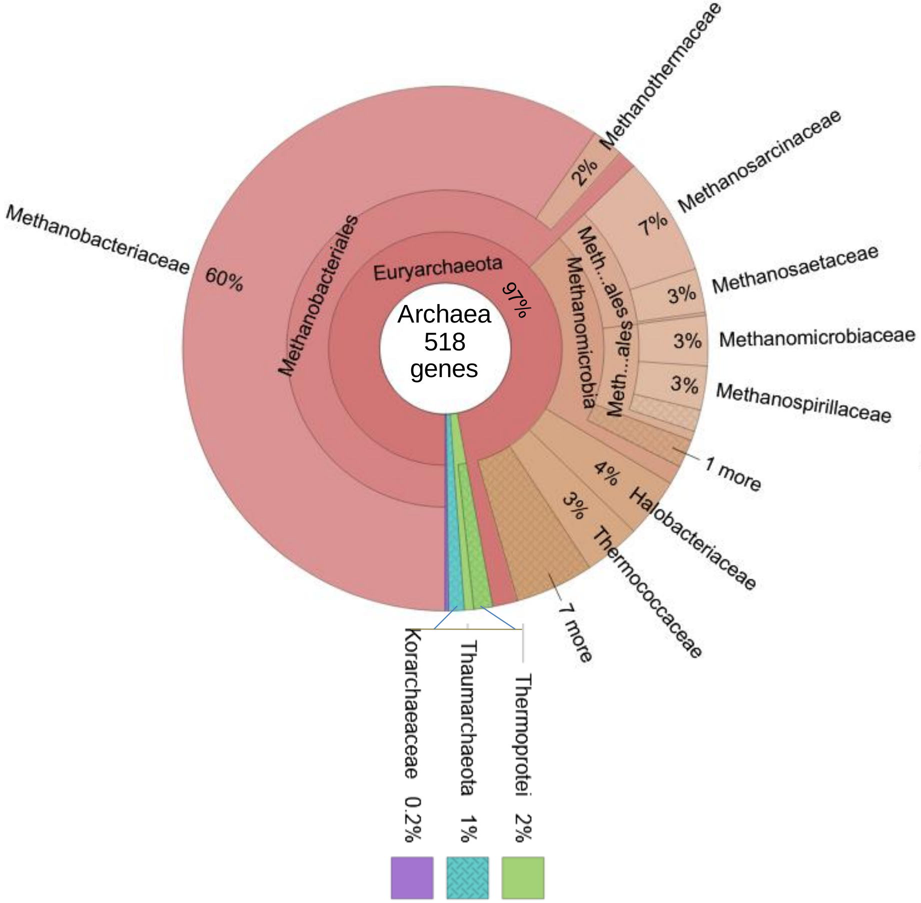

Total archaeal composition that were assigned into phylum level

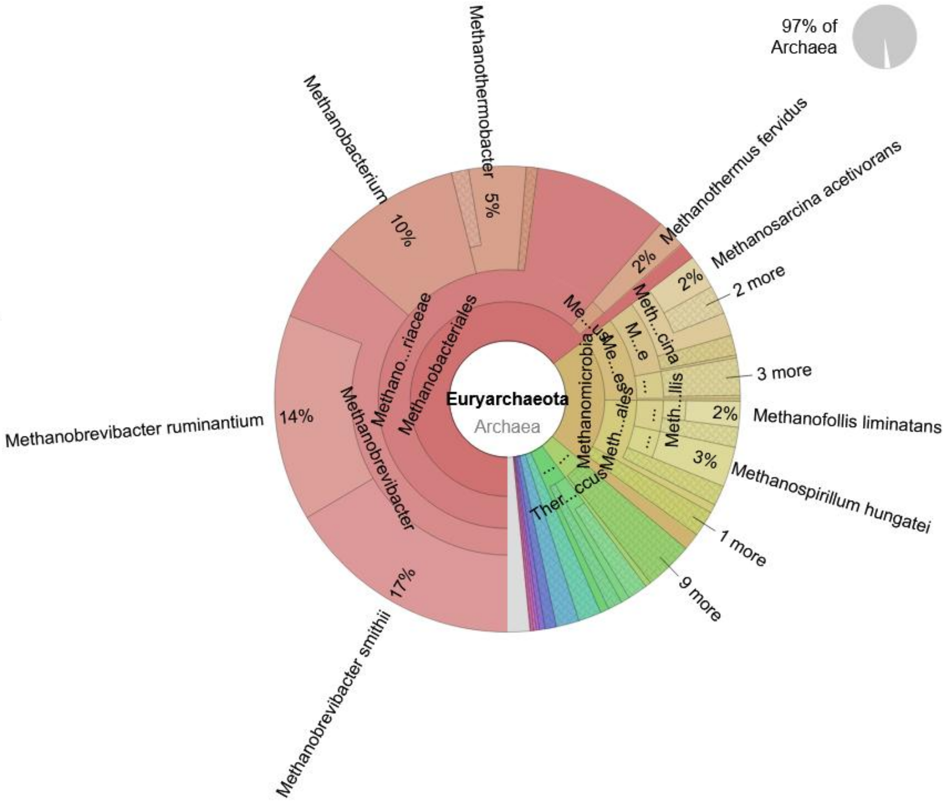

Total Euryarchaeota composition

Figure S3. The compositional contribution of bacteria harbouring genes coding for CAZymes in the gut of C. gestroi

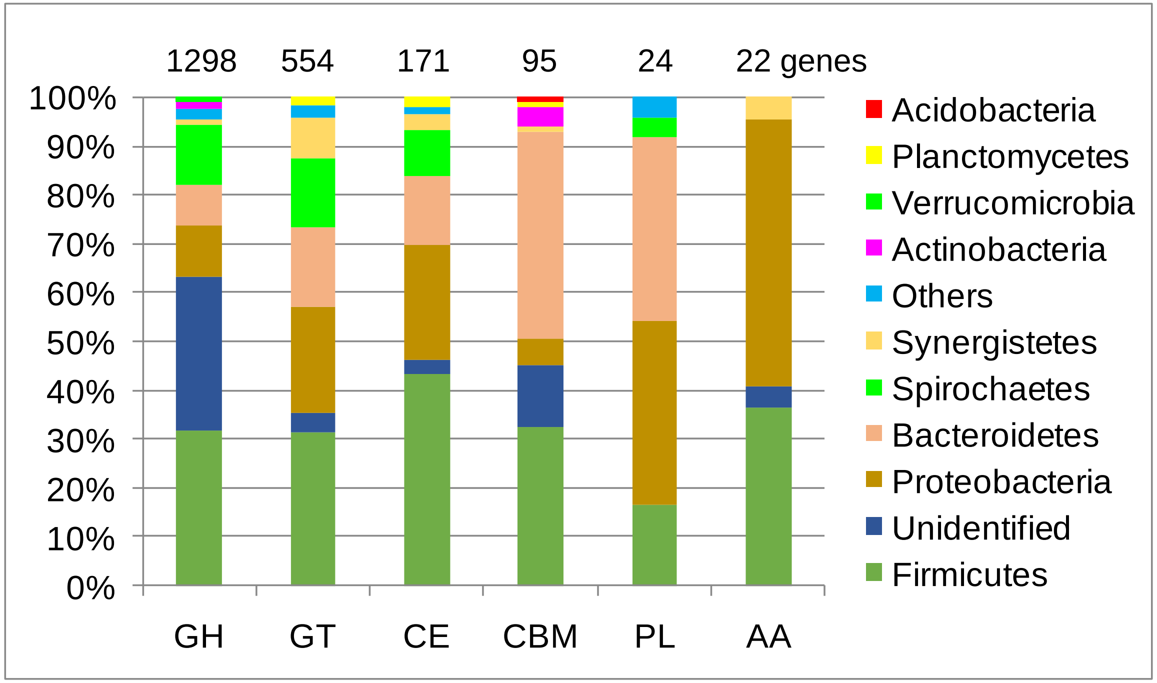

Phylum

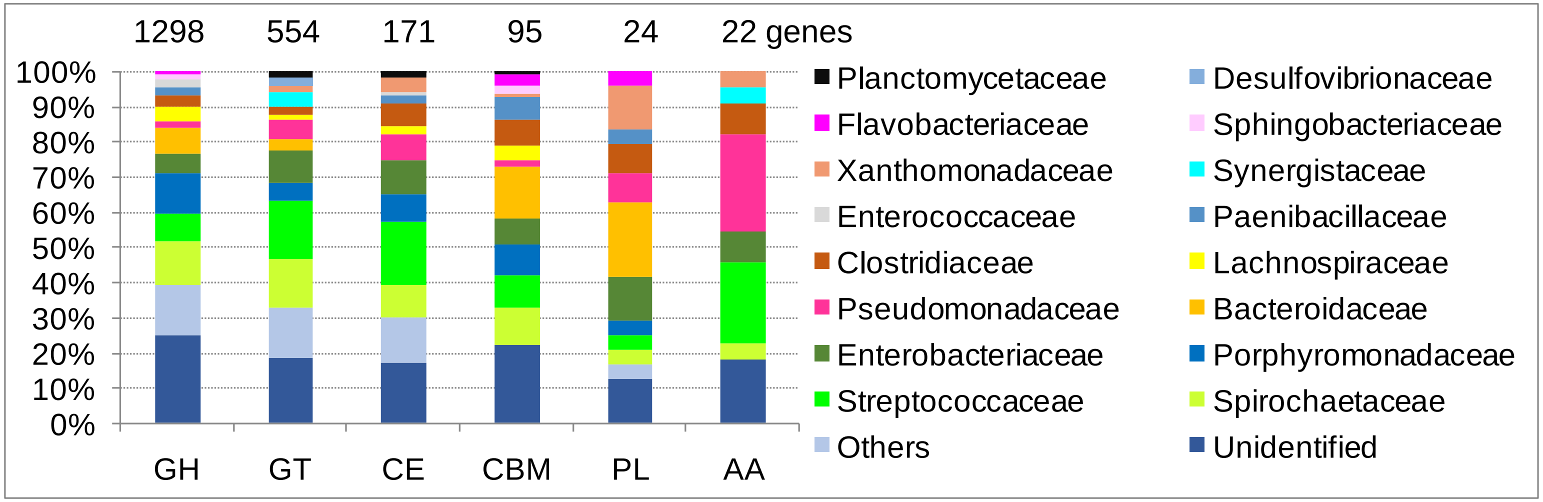

Family

Figure S3. (Continued)

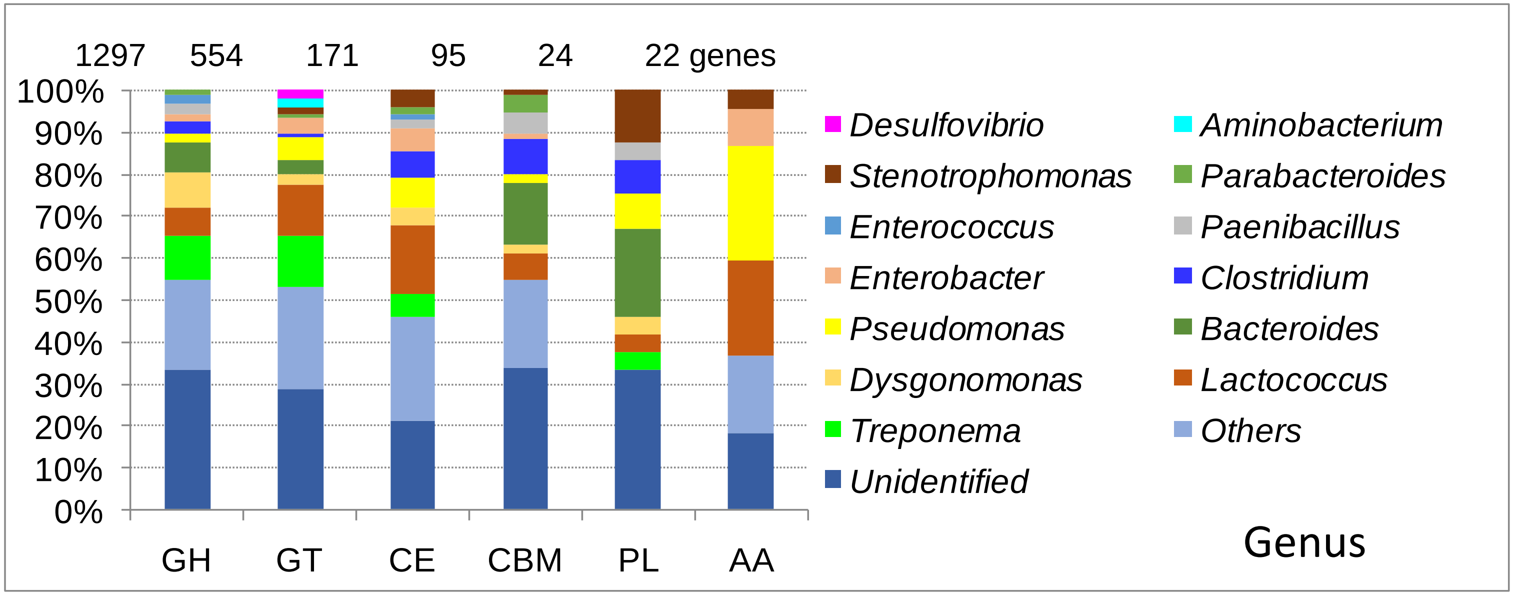

	GT		GH		CBM		CE		PL		AA	
	Genes	%	Genes	%	Genes	%	Genes	%			Genes	%
Unidentified species	263	47.5	648	49.9	49	51.6	71	41.5	8	33.3	12	54.5
Identified species	291	52.5	650	50.1	46	48.4	100	58.5	16	66.7	10	45.5
Total	554	100.0	1298	100.0	95	100.0	171	100.0	24	100	22	100.0

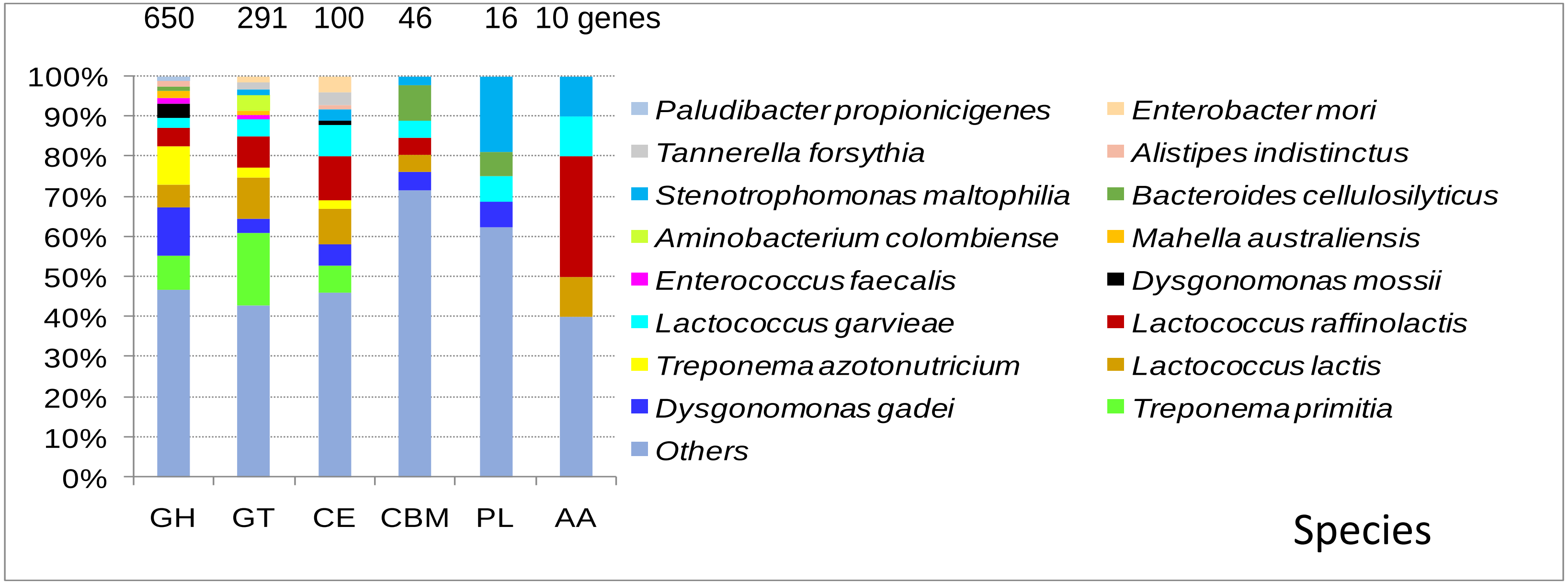

Figure S4. The compositional contribution of bacterial species harboring genes related in antibiotics' synthesis in the gut of C. gestroi

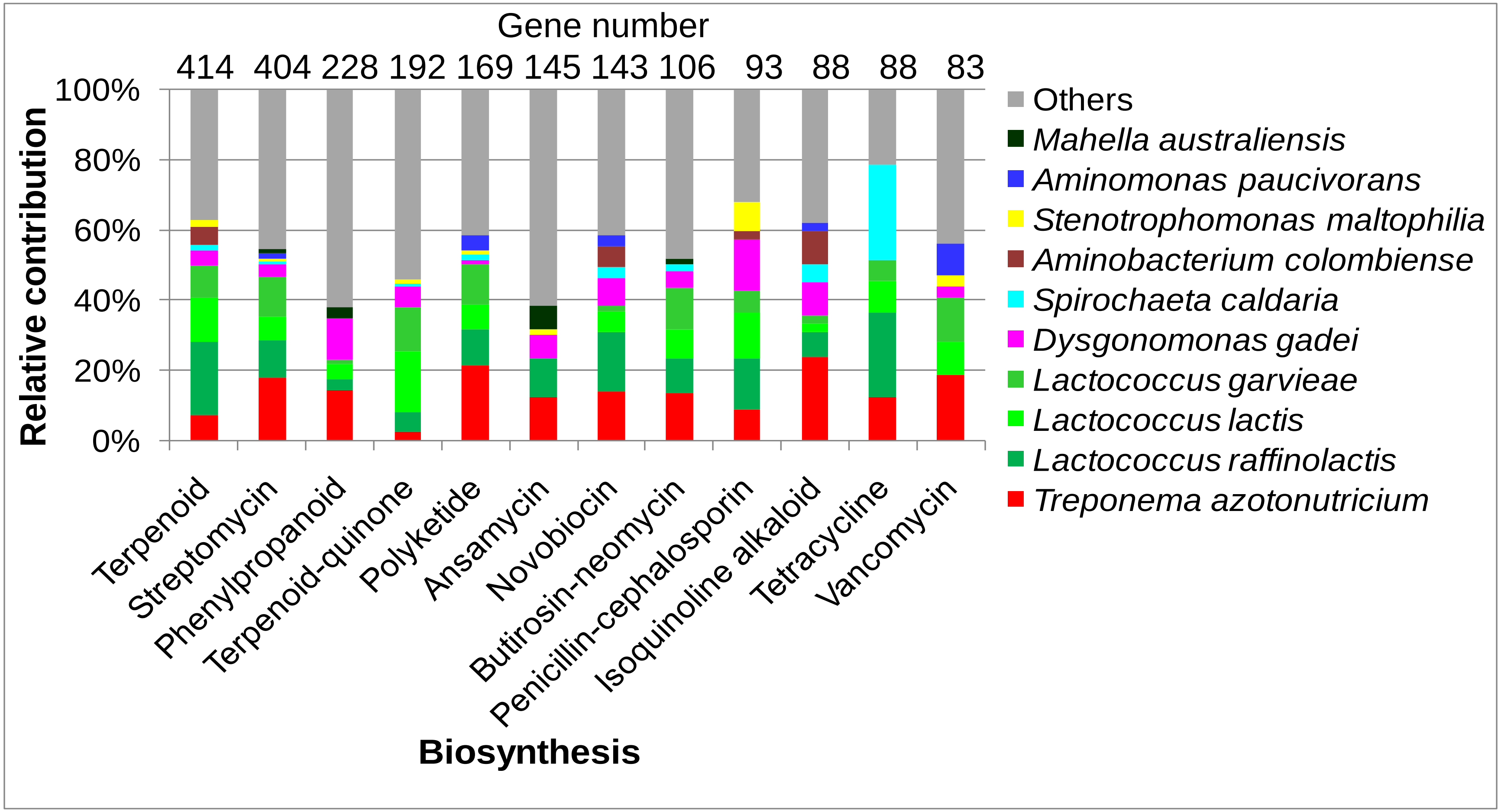

Figure S5. Comparison of bacterial diversity at phylum level of bacteria free-living in C. gestroi guts in this study with the average diversity of bacteria in 74 samples of termite (Arora et al., 2022)

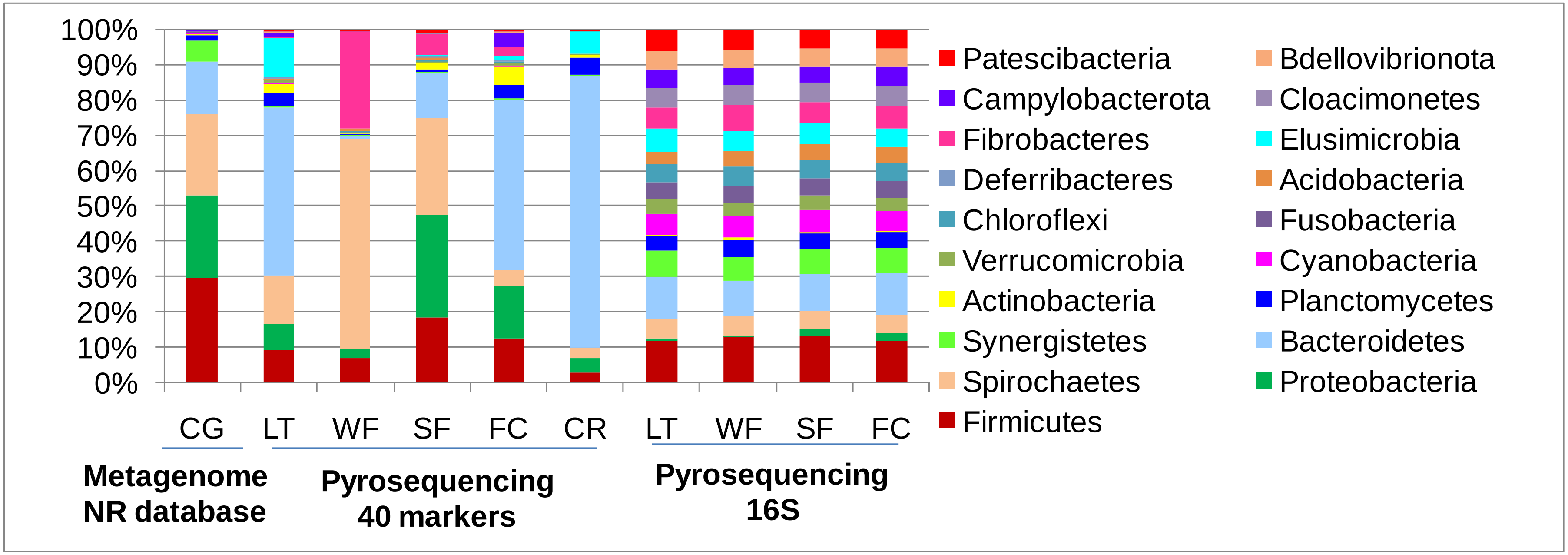

C. gestroi (CG, 1 sample), coakroach (1 sample), lower termites (LT, 25 samples), non-Macrotermitinae wood-feeding Termitidae (WF, 19 samples), fungal-cultivating termites (FC, 5 samples) and soil-feeding termites (SF, 25 samples).

Figure S6. Relative abundance of CAZymes found in metagenomic DNA data of bacteria free-living in C. gestroi gut and bacteria in cockroach gut, 129 termite species gut that belonging to four groups (Arora et al, 2022)

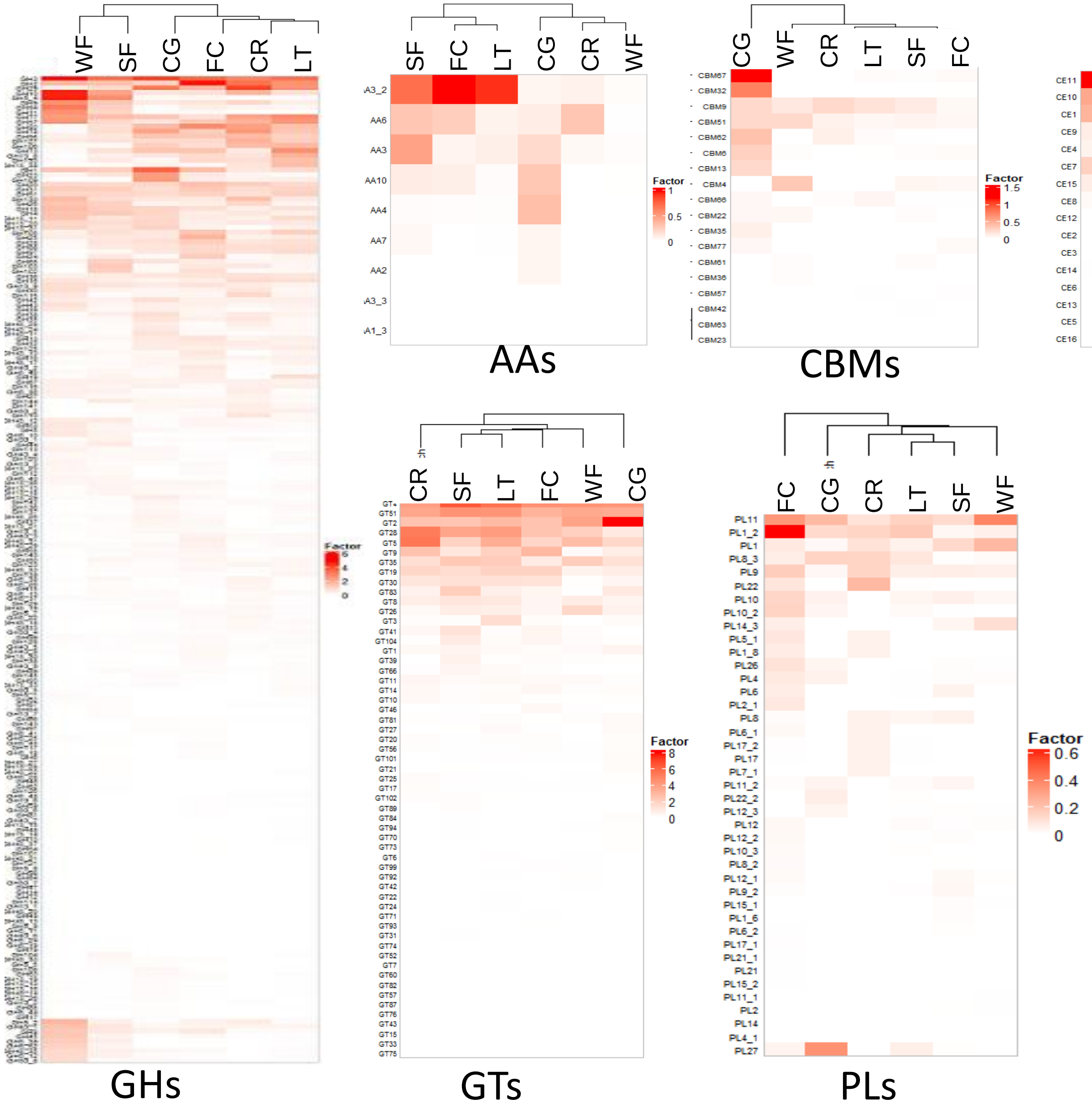

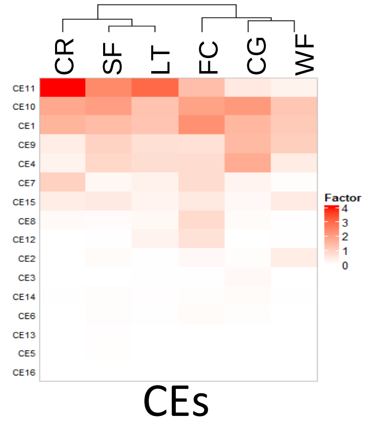

Samples	Number
Coptotermes gestroi (in this study)	1
Lower termites (LT)	45
Cockroach	1
Non-Macrotermitinae wood-feeding Termitidae (WF)	32
Fungal-cultivating termites (FC)	9
Soil-feeding termites (SF)	42
